# An Economical Method for Production of ^2^H,^13^CH_3_-Threonine for Solution NMR Studies of Large Protein Complexes: Application to the 670 kDa Proteasome

**DOI:** 10.1371/journal.pone.0043725

**Published:** 2012-09-11

**Authors:** Algirdas Velyvis, Amy M. Ruschak, Lewis E. Kay

**Affiliations:** 1 Departments of Molecular Genetics, Biochemistry, and Chemistry, University of Toronto, Toronto, Ontario, Canada; 2 Program in Molecular Structure and Function, Hospital for Sick Children, Toronto, Ontario, Canada; Spanish National Cancer Center, Spain

## Abstract

NMR studies of very high molecular weight protein complexes have been greatly facilitated through the development of labeling strategies whereby ^13^CH_3_ methyl groups are introduced into highly deuterated proteins. Robust and cost-effective labeling methods are well established for all methyl containing amino acids with the exception of Thr. Here we describe an inexpensive biosynthetic strategy for the production of L-[α-^2^H; β−^2^H;γ-^13^C]-Thr that can then be directly added during protein expression to produce highly deuterated proteins with Thr methyl group probes of structure and dynamics. These reporters are particularly valuable, because unlike other methyl containing amino acids, Thr residues are localized predominantly to the surfaces of proteins, have unique hydrogen bonding capabilities, have a higher propensity to be found at protein nucleic acid interfaces and can play important roles in signaling pathways through phosphorylation. The utility of the labeling methodology is demonstrated with an application to the 670 kDa proteasome core particle, where high quality Thr ^13^C,^1^H correlation spectra are obtained that could not be generated from samples prepared with commercially available U-[^13^C,^1^H]-Thr.

## Introduction

Many of the advances in protein NMR spectroscopy can be directly traced to the development of isotope labeling strategies that have substantially increased the range of biomolecular systems that can be explored [1]–[6]. For example, applications to very high molecular weight complexes have benefited significantly from the preparation of highly deuterated molecules where the relaxation times of the remaining NMR probes, typically backbone amide moieties or side-chain methyl groups, are significantly increased [7], [8]. Concomitant with the emergence of these important labeling approaches has been the advancement of new NMR experiments that exploit the labeling in ways that permit the recording of spectra of both increased sensitivity and resolution [9], [10].

Over the past 15 years our laboratory has developed a strategy for studying high molecular weight protein complexes that involves ^13^CH_3_ labeling of Ile (δ1 or γ2), Leu and Val methyl positions in an otherwise highly deuterated ^12^C background [11]–[13]. Spectra are recorded that make use of a methyl-TROSY effect that results in significant line-narrowing [14]. Applications of this methodology to a large number of systems have now been reported [15]–[27], along with schemes for extending the labeling to Ala [21], [28] and Met [16], [29], [30] methyl positions or for stereospecific incorporation of methyl labels at either pro*R* or pro*S* positions of Leu and Val side-chains [31]. More recently an approach for placement of methyl groups at positions of interest has been introduced involving substitution of the native residue with Cys and subsequently reacting with ^13^C-methyl-methanethiosulfonate (^13^C-MMTS) [32].

Ile, Leu, Val comprise approximately 20% of the amino acids in a ‘typical’ protein, and Ala, Met approximately 10% and 2%, respectively [33]. It is thus expected that in many cases these residues, in various combinations, will provide ‘excellent coverage’ of the protein in the sense that they will be found in regions that contribute in important ways to the structure or dynamics of the molecule studied. However, as pointed out by Rule and coworkers [34] these residues are under-represented at protein-nucleic acid interfaces. Moreover, Ile, Leu, Val and Met are predominantly partitioned inside proteins, while Ala has a small preference for the interior as well [35]. Thus, these residues are not effective probes of protein surfaces. The one remaining methyl containing residue, Thr, has both a much higher relative propensity for placement at protein-nucleic acid interfaces [34] and, not surprisingly, also a higher composition on protein surfaces relative to the interior [35]. In addition, of all methyl-containing residues the hydrogen bonding functionality of the Thr side-chain is unique. Finally, like other amino acids, Thr residues can play critically important roles in protein function, such as is the case for the proteasome, a Thr protease [36], [37], that forms the basis of a large research effort in our laboratory (see below).

Rule and coworkers have recently introduced a method to label methyl groups of Thr in recombinant proteins with the ^13^CHD_2_ isotopomer by using 2-^13^C-glycerol and bicarbonate during bacterial growth in D_2_O [34]. This methodology is most certainly applicable to small-intermediate sized proteins, as illustrated by this group. However, applications to very large complexes with aggregate molecular weights in the hundreds of kDa will be compromised by an extent of labeling of only approximately 25%, by the fact that fully protonated glycerol is used as the carbon source so that protons will be introduced in positions other than methyl groups and because it is not possible to generate the ^13^CH_3_ methyl isomer while still retaining a high level of protein deuteration. Kainosho and coworkers have developed an elegant approach for protein labeling (termed the SAIL method) in which amino-acids with the desired labeling pattern are prepared via organic chemistry and then added, typically to a cell-free protein synthesis system, to produce suitably labeled proteins [4]. Recently, Thr labeled with ^2^H and ^13^C at the Cβ (C3) position was prepared for studies of side-chain hydrogen exchange [38]. Unfortunately, the cost of such labeled amino acids can be high. Moreover, Thr with the labeling pattern that we seek here for studies of supra-molecular protein complexes (see below) is not commercially available presently from SAIL Technologies Inc., suppliers of SAIL amino acids.

With this in mind we describe here the biosynthesis of L-[α-^2^H;β−^2^H;γ-^13^C] Thr (U-[^2^H],Thr-γ^2^[^13^CH_3_]) starting from the relatively inexpensive precursor, ^13^C formaldehyde (60% yield). All of the enzymes necessary for the synthesis have been expressed and purified and are available upon request. The utility of the methodology is illustrated with an application to the 20S proteasome core particle from *T. acidophilum*, 670 kDa, that plays an integral role in cellular homeostasis [37]. We show that all 15 of the expected correlations from Thr methyl groups of the labeled β-subunit of the enzyme are present in ^13^C,^1^H NMR correlation maps; notably only a modest fraction of the expected peaks could be observed when commercially available, uniformly ^13^C and fully protonated Thr was used as a precursor. Assignments of the majority of the Thr resonances could be made from NOESY data sets correlating previously assigned Ile, Leu, Val methyls with Thr methyl groups. With the development of a cost effective strategy for the biosynthesis of U-[^2^H],Thr-γ2 [^13^CH_3_] it is now possible to generate highly deuterated samples of very high molecular weight protein complexes with Thr probes of structure and dynamics.

## Results and Discussion

### Biosynthesis of U-[^2^H],Thr-γ2[^13^CH_3_]

**Figure 1:**
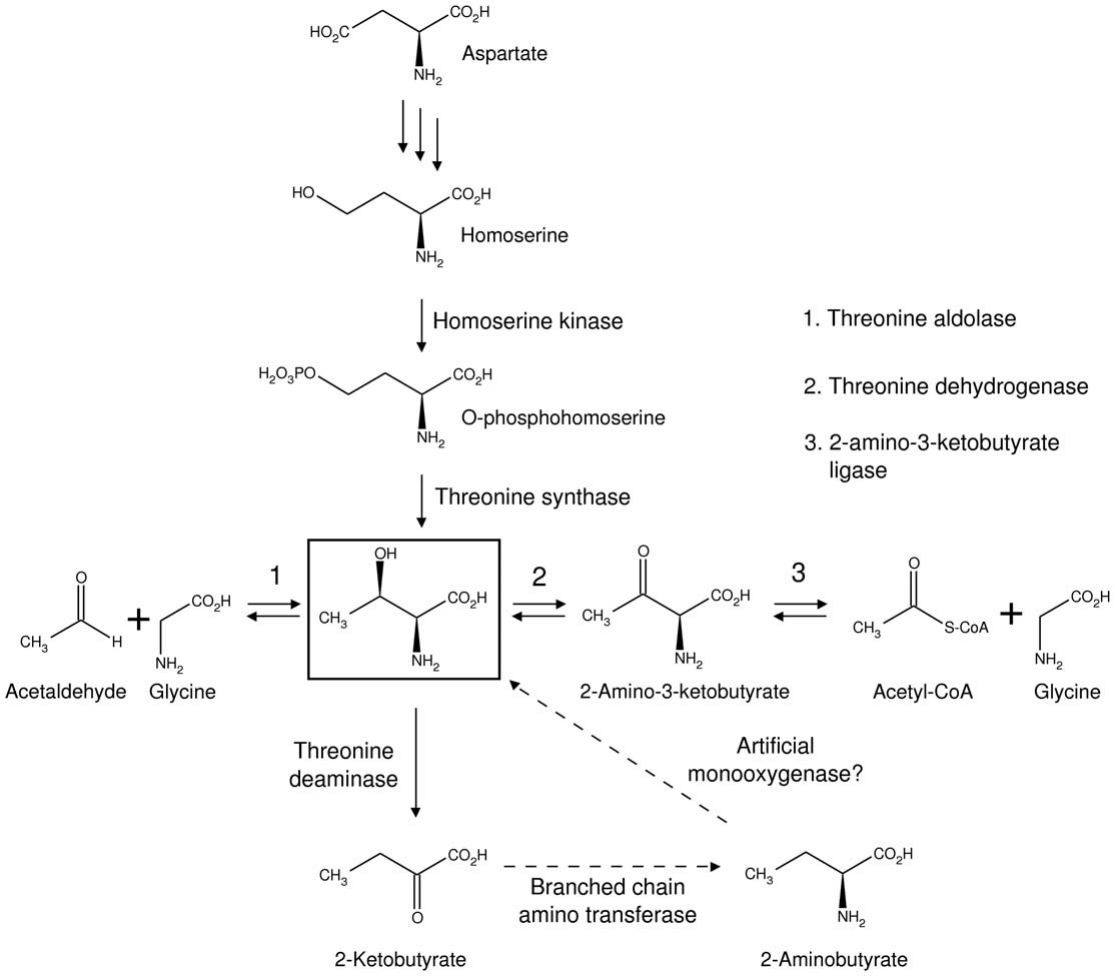
Metabolism of Thr starting from Asp. The structure of Thr is highlighted in the box and the Thr to Asp pathway is along the vertical. Also shown are pathways that potentially can be exploited to synthesize Thr and the enzymes that are involved (horizontal). Dashed lines (bottom) indicate the non-physiological conversion of 2-ketobutyrate into L-2-aminobutyrate and then into Thr.

Prior to the development of a strategy for the production of U-[^2^H],Thr-γ2[^13^CH_3_] labeled proteins we first examined the biosynthetic pathway of Thr to evaluate whether an appropriately labeled precursor of this amino acid could be generated that would then be added during protein expression. Such an approach has been used successfully in the development of 2-ketyobutyrate and 2-ketovalarate, precursors for Ile(δ1) and Leu/Val respectively [11], [12], that are used to produce U-[^2^H], Ile-δ1[^13^CH_3_]- or U-[^2^H], Leu,Val-[^13^CH_3_,^12^CD_3_]-labeled proteins [13] and more recently for the generation of precursors that result in proteins with Ile γ2 CH_3_ labeling [39] or stereospecific CH_3_ methyl group labeling of Leu and Val residues [31]. Figure 1 illustrates the biosynthesis of Thr (boxed compound) starting from Asp. Of particular interest is that the final step, catalyzed by threonine synthase (TS), involves addition of a solvent hydrogen to the 4 position of O-phosphohomoserine. Because protein expression is carried out in D_2_O, a prerequisite for the generation of highly deuterated proteins that is required for NMR studies of very high molecular weight complexes, the methyl group of the Thr so produced will contain at least one deuteron. It has been shown previously that the optimum labeling strategy for methyl groups is ^13^CH_3_ so that a methyl-TROSY effect can be exploited [40], [41]. Addition of a precursor in the Thr synthesis pathway is thus not a viable option for the production of proteins with U-[^2^H],Thr-γ2[^13^CH_3_].

An alternative strategy is one where a precursor is introduced to a cell line where the main, TS-dependent, biosynthetic pathway is inactivated. In this manner Thr could be assembled from glycine and a ^13^CH_3_-labeled acetyl fragment (either acetaldehyde or acetyl-CoA) via threonine aldolase or 2-amino-3-ketobutyrate ligase, Figure 1. Neglecting questions relating to the efficiency of such a scheme, the central role of acetyl-CoA in cellular metabolism will inevitably result in any acetyl fragment labeling other amino acids with ^1^H and ^13^C.

We therefore turned to an approach whereby U-[^2^H],Thr-γ2[^13^CH_3_] would be added to growth media during protein expression. Several schemes for the ‘in-vitro’, enzymatic synthesis of Thr are possible, as indicated in Figure 1. First, condensation of 1-^2^H,2-^13^C acetaldehyde with deuterated glycine via threonine aldolase [42], [43] generates Thr with the desired labeling pattern. However, acetaldehyde labeled in this manner is expensive ($5500/g, minimum order of 3 g) and both L-Thr (2*S*,3*R*) and L-allo-Thr (2*S*,3*S*) are produced, necessitating separation. Second, Thr can be produced via a pathway involving steps catalyzed by 2-amino-3-ketobutyrate ligase and threonine dehydrogenase [44], with ^13^C-labeled sodium acetate serving as a source of acetyl-CoA (Figure 1). However, the production of large quantities of Thr is likely to be compromised because (i) the intermediate 2-amino-3-ketobutyrate has a half-life estimated to be less than 1 minute [45] or 10 minutes [46] at pH 7, with spontaneous decarboxylation diverting a large fraction of the isotopically labeled material into aminoacetone [44] and (ii) 2-amino-3-ketobutyrate ligase also possesses some threonine aldolase activity [47] which would destroy the desired product (Thr) in a coupled enzymatic reaction. A third approach is also suggested in Figure 1, starting from 2-ketobutyrate (2KB, ^13^CH_3_^12^CD_2_COCOONa), a well-established and inexpensive commercially available precursor for labeling of Ile δ1 positions with ^1^H and ^13^C [11]. 2KB can be transaminated into L-2-aminobutyrate either by branched chain aminotransferase or an engineered dehydrogenase [48]. If an enzyme could be found that oxidizes 2-aminobutyrate to Thr it would enable conversion of 2KB into the desired labeled product. While it is known that monooxygenases of the cytochrome P450 family can hydroxylate aliphatic carbon positions [49], [50] and indeed P450BM3 which is specific for fatty acids can be engineered to hydroxylate butyrate [51], to the best of our knowledge a P450 that produces Thr has not been reported.

**Figure 2:**
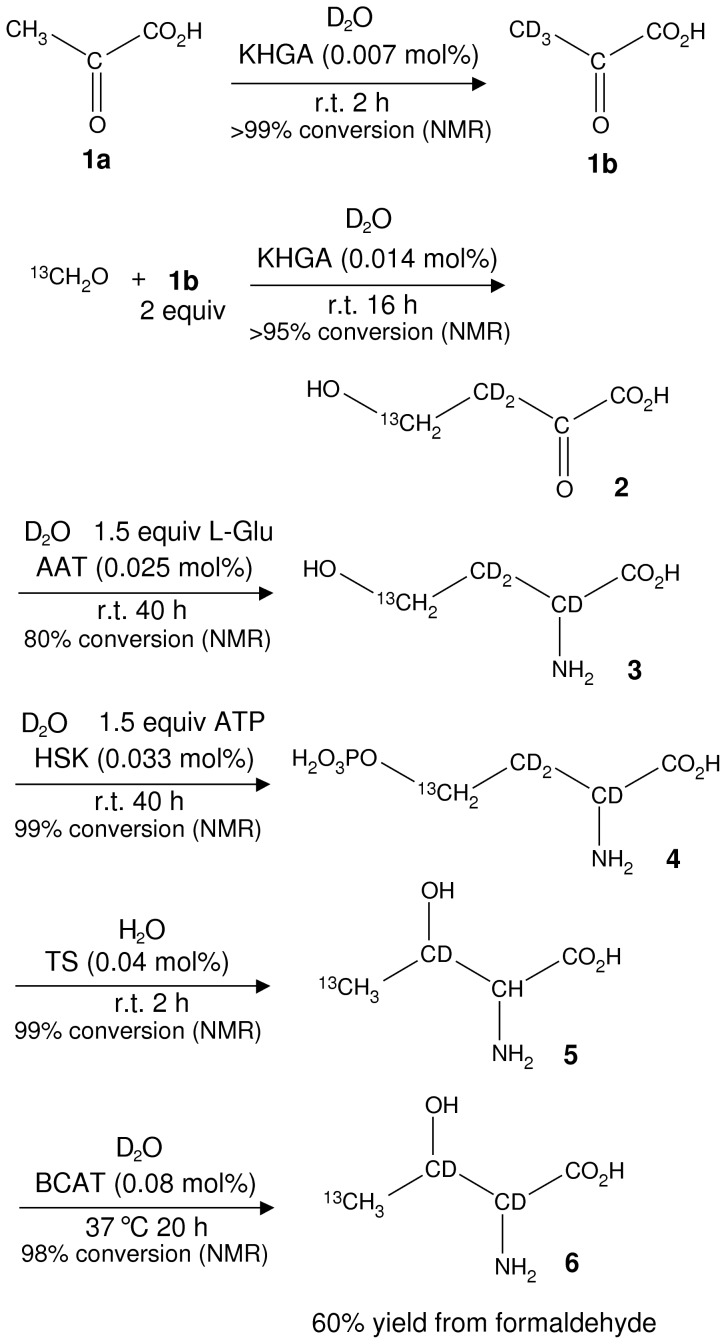
Scheme used for the biosynthesis of U-[^2^H],Thr-γ2[^13^CH_3_], 6. Enzyme name abbreviations: KHGA = 2-keto-4-hydroxyglutarate aldolase, AAT = aspartate aminotransferase, HSK = homoserine kinase, TS = threonine synthase, BCAT = branched-chain-amino-acid aminotransferase.

The strategy that we have adopted, therefore, is to synthesize Thr by ‘reproducing’ the major Thr biosynthesis pathway in the ‘test-tube’, Figure 1 top, whereby Asp is converted into homoserine in 3 steps and then to Thr by homoserine kinase (HSK) and threonine synthase. A simplification to the path taken by nature involves generating homoserine **3** via transamination of the keto acid, 2-keto-4-hydroxybutyrate (KHB, **2**), as indicated in Figure 2, which summarizes the Thr biosynthesis route used here. The first critical step is condensation of ^2^H, pyruvate **1b** with ^13^C-formaldehyde, catalyzed via the enzyme 2-keto-4-hydroxyglutarate aldolase [52], [53] (KHGA, also known as 2-keto-3-deoxy-6-phosphogluconate aldolase), a member of the pyruvate aldolase family that has been used in biocatalysis previously [54]. The product of this reaction (referred to as reaction 1 in what follows) is KHB **2**, in which the entire carbon chain for Thr is assembled with the correct deuteration and ^13^C labeling pattern at C3 and C4. Importantly, the ^13^C-formaldehyde precursor is available commercially and is relatively inexpensive, with a list price for ^13^C-paraformaldehyde of $720/gram that compares very favorably to the cost of a custom synthesis of 1-^2^H,2-^13^C acetaldehyde ($16,500 for a minimum order of 3 grams). Additionally, ^2^H-pyruvate, **1b**, is readily generated from pyruvate **1a** via incubation with catalytic amounts of KHGA in D_2_O based buffer for 1 hour at pH 7.5. The equilibrium constant for the production of **2** from **1b** is 250 M^−1^ [52] so that by adding excess pyruvate the reaction can be made to go to completion; in a mixture starting as 0.1 M formaldehyde and 0.2 M pyruvate 96% of the starting formaldehyde is predicted to be converted to KHB. Indeed, when depolymerized ^13^C-paraformaldehyde (0.11 M) is mixed with a two-fold excess of pyruvate in the presence of KHGA the aldehyde signals in ^1^H NMR spectra disappear, while those from KHB appear, with greater than 95% conversion (see Supporting Information S1). Moreover, when the reaction is carried out in 99.9% D_2_O only the C4 attached hydrogens in **2** are observed in spectra, as expected (Supporting Information S1). Upon attainment of equilibrium (within several hours when 0.014 mole percent of KHGA is used at room temperature) the reaction solution is filtered to remove the biocatalyst and used in the next step without further purification.

The transamination of KHB **2** to homoserine **3** can reportedly be catalyzed by aspartate and alanine aminotransferases [52]. Aspartate aminotransferase (AAT, see Supporting Information S1) was the most efficient of several enzymes tested with 60% conversion to homoserine in 3 hours using 1.5 equivalents of Glu as the amino donor. A point of concern regarding transamination reactions is their reversibility since ‘similar’ types of compounds are interconverted (amino acid+keto acid→keto acid+amino acid). A large excess of Glu may be required to drive the reaction of interest to completion, unless it can be coupled to an essentially irreversible downstream reaction, such as phosphorylation by ATP. For this reason it is beneficial to combine transamination of **2** and phosphorylation of **3** into a single procedure (we designate this “reaction 2–3”). NMR analysis indicates that reaction 2–3 stops after about 40 hours with approximately 20% of the original 0.1 M KHB **2** remaining and only 1% homoserine **3** (Supporting Information S1). At intermediate times we observed much higher levels of homoserine as illustrated in Supporting Information S1, where the reaction on fully protonated precursors in H_2_O solvent is shown. The simplest explanation for these levels of conversion is that transamination of **2** proceeds rapidly but that AAT has a high K_M_ for KHB (so that the reaction effectively terminates at a low concentration of KHB, 20 mM). In contrast, because homoserine **3** is the canonical substrate for HSK it has a much higher affinity, K_M_<1 mM [55], but the kinetics of the HSK reaction are slower than for the AAT catalyzed step (hence the observation of the homoserine intermediate).

O-phospho-L-homoserine, OPHS **4**, was subsequently purified from the 2–3 reaction mixture using ion exchange chromatography [56] (Supporting Information S1), removing any protonaceous organic contaminants such as 2-ketoglutarate, glutamate and possibly ADP/ATP from the transamination step (Supporting Information S1). Purified OPHS (∼0.1 M, also containing up to 1 M NaCl from the ion exchange eluent) is efficiently converted by TS in H_2_O to Thr **5** and one equivalent of inorganic phosphate. However, an inevitable consequence of the TS-catalyzed reaction in H_2_O is protonation of **5** at C2. This necessitates a final H/D exchange step. Water is evaporated, the resultant Thr **5**, phosphate and NaCl mixture dissolved in 99.9% D_2_O and the enzyme, BCAT (branched-chain-amino-acid aminotransferase) added to catalyze H/D exchange, producing Thr with the desired labeling pattern, **6**.

**Figure 3:**
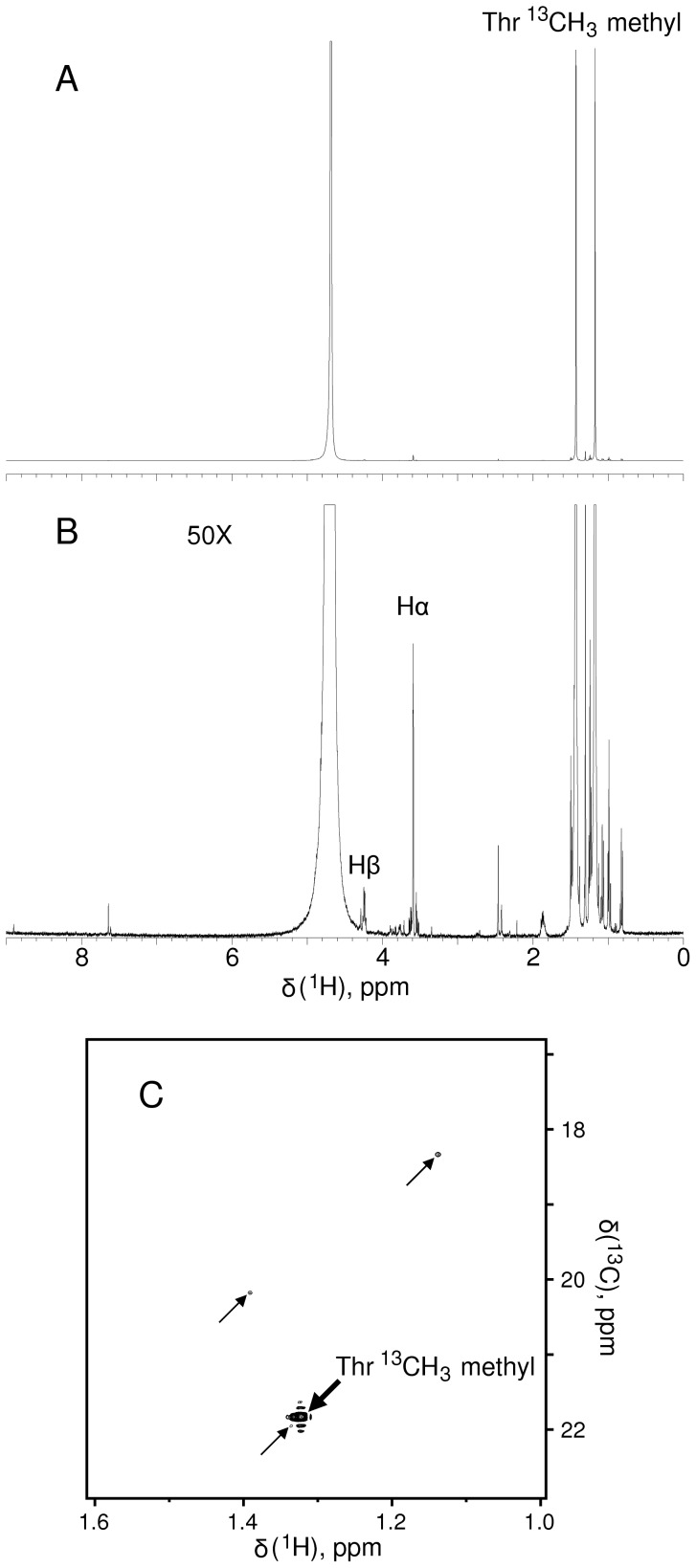
NMR spectra of L-[α-^2^H;β−^2^H;γ-^13^C]-Thr, 6. (A) ^1^H NMR spectrum with vertical scale adjusted to fit entire methyl doublet. (B) Same spectrum as in A with a 50-fold enhanced vertical scale. (C) ^13^C,^1^H HSQC spectrum of the same sample, **6**. Contour level is set to 0.5% of the Thr methyl signal. The only 3 other peaks (all under 1% of the intensity of the Thr methyl) derive from impurities and are indicated with arrowheads. Approximately 2% residual protonation remains at the α position and much less at β.

NMR spectra of the final product **6** are shown in Figure 3, establishing excellent purity, a high degree of perdeuteration at C2 and C3 and low amounts of contaminants. After filtering out the last biocatalyst the D_2_O solution containing 78 mM Thr **6**, ∼80 mM sodium phosphate, ∼1 M NaCl, and ∼0.5 mM pyridoxal phosphate can be used directly for recombinant protein labeling or stored in frozen form. Two batches of Thr were synthesized in this manner and in each case 2.5 mmol amino acid was produced (300 mg) from a starting amount of 4.1 mmol (127 mg) paraformaldehyde, corresponding to a yield of 60%.

### Testing labeling strategies

In order to establish the efficiency of Thr labeling in proteins and to determine both the amount of precursor to add and whether other compounds should be included simultaneously in the growth medium we have prepared several samples of U-[^13^C,^1^H] labeled Abp1p SH3 domain where ^12^C labeled precursors such as Thr, 2KB or 2-ketoisovalerate (2KIV) have been added approximately one hour prior to the induction of protein overexpression (see Supporting Information S1) in standard BL21(DE3) *E. coli* cells. Because of the small size of the domain (∼60 amino acids) it is straightforward to quantify peak intensities accurately in a series of constant time ^13^C,^1^H HSQC data sets. Decreases in peak intensities reflect incorporation of (unlabeled) precursor, providing a gauge of the extent of both desired and undesired labeling.

**Figure 4:**
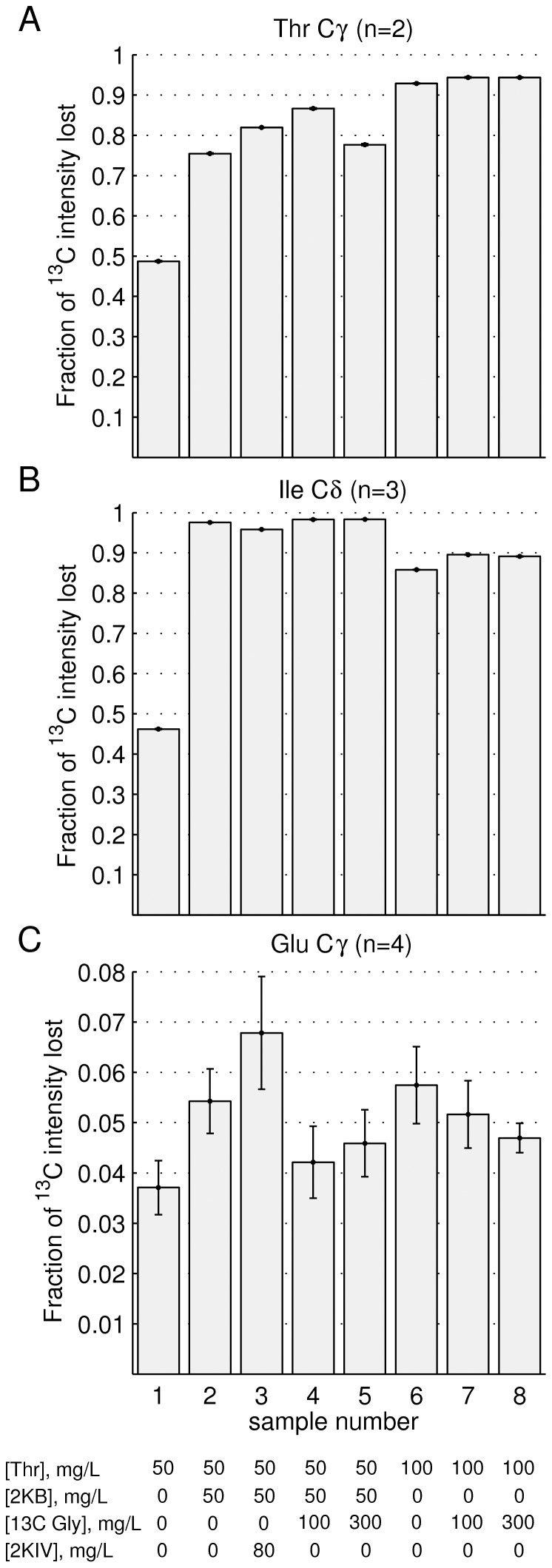
Efficacy of Thr labeling in proteins. Fraction of ^13^C intensity lost at Thr Cγ (A), Ile Cδ1 (B) and Glu Cγ (C) positions of a U-[^13^C,^1^H] labeled Abp1p SH3 domain sample where ^12^C labeled precursors such as Thr, 2-ketobutyrate or 2-ketoisovalerate (2KIV) are added in the amounts indicated at the bottom of the figure prior to induction of protein expression. The value of ‘n’ corresponds to the number of residues of a particular type that are averaged over in each measurement, with the error bars denoting the complete range of values obtained. Details are in Supporting Information S1.

Figure 4 displays the fraction of ^13^C intensity lost at Thr Cγ (A), Ile Cδ1 (B) and Glu Cγ (C) positions as a function of added precursor. The nature of the precursor(s) and the amounts added are indicated along the x-axis. Addition of 50 mg/L Thr leads to ∼50% labeling at both Thr γ2 and Ile δ1 positions (sample 1). Labeling of Ile is not unexpected since Thr is a precursor of Ile biosynthesis [57]. In order to avoid it Rule and coworkers add perdeuterated Ile to their growth media [34], similar to Ayala *et al* in their strategy for labeling Ala methyl groups [28]. Here we have taken an opposite approach. Because cross peaks for Ile residues fall in an isolated region of the ^13^C,^1^H correlation map and are among the most well resolved of all methyl types we prefer to include Ile labeling in all of our Thr samples (and often also Leu, Val). The advantages are two-fold. First, the increased proton density in the protein, relative to Thr only ^13^CH_3_ methyl samples, reduces ^1^H T_1_ values leading to sensitivity improvements. Second, addition of the inexpensive 2KB (Ile) precursor ensures that the added U-[^2^H],Thr-γ2[^13^CH_3_] is not diverted to Ile, leading to higher incorporation of the desired Thr label. Thus, by adding both 50 mg/mL Thr and 50 mg/mL 2KB the fractional labeling increases to 75% and >95% for Thr γ2 and Ile δ1, respectively. Alternatively, addition of larger quantities of Thr (100 mg, sample 6) ensures that, despite the diversion of this compound to Ile, there is still a sufficient amount to achieve high labeling of Thr Cγ2 (>90%). Increasing the quantities of added Thr does lead to some isotope scrambling, however. ^13^C,^1^H HSQC spectra of the SH3 domain indicate that when 100 mg/L of ^1^H,^12^C Thr is added to the growth medium there is an approximately 40% reduction in peaks from Gly (Supporting Information S1). Note that Thr can be converted to Acetyl-CoA and Gly via the threonine dehydrogenase pathway [44], as shown in Figure 1. In a ‘real’ sample where U-[^2^H],Thr-γ2[^13^CH_3_] is added to a D_2_O based growth the Gly produced is fully deuterated so that additional protons are not added to the protein. However, this pathway does dilute the Thr precursor. Moreover, the ^13^CH_3_ acetyl-CoA so produced is a precursor in the synthesis of Glu, with the Hγ and Cγ positions derived from the methyl group. The level of incorporation at these sites does not appear to be high, however, Figure 4C. Addition of ^13^C-Gly during the production of SH3 domain samples generated from unlabeled Thr and 2KB does reduce the extent of scrambling from Thr to Gly and slightly to Glu. Importantly, the extent of Thr labeling increases to close to 90% from 75% (compare samples 2 and 4), indicating that diversion of Thr into undesirable pathways is at least partially inhibited. Given that perdeuterated glycine is inexpensive we suggest 50 mg/L labeled U-[^2^H],Thr-γ2[^13^CH_3_], 50 mg/L labeled 2KB (^13^CH_3_CD_2_COCOONa) and 100 mg/L d_5_-glycine as an optimal combination for production of highly deuterated proteins labeled with ^13^CH_3_ at Thr and Ile (δ1) methyl positions.

### An application to the proteasome core particle

**Figure 5:**
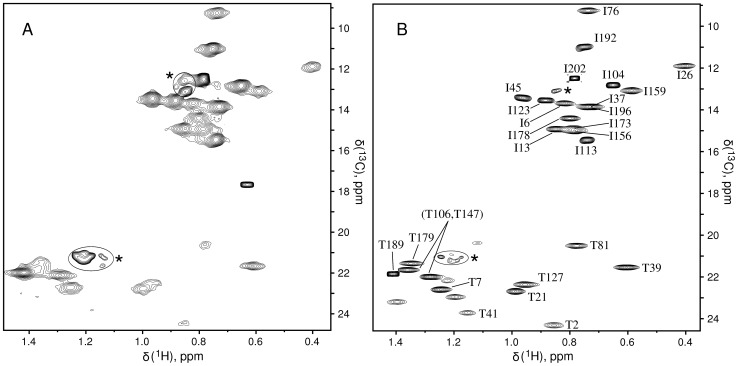
NMR spectroscopy of Thr-γ2[^13^CH_3_]-labeled proteins. (A) ^13^C,^1^H HMQC spectrum of U-[^2^H] α_7_β_7_β_7_α_7_ prepared with U-[^13^C,^1^H]-Thr (labeled in the β subunit, 200 µM subunit concentration) recorded in 18 hours, 70°C, 18.8T. ^13^Cβ Thr band selective adiabatic decoupling [65] was applied to narrow the Thr ^13^Cγ resonance lines. (B) ^13^C,^1^H HMQC spectrum of 0.54 mM (subunit concentration) U-[^2^H], Ile-δ1[^13^CH_3_], Thr-γ2[^13^CH_3_] α_7_β_7_β_7_α_7_ (labeled in the β subunit) recorded in 2 hours at 70°C, 18.8T. Partial assignments are indicated; stars denote degradation product peaks. The difference in acquisition times is intended to compensate for the concentration differences. It is clear, however, that much higher quality spectra are derived from samples prepared with the current labeling scheme.

The 20S proteasome core particle (CP) is a 670 kDa barrel-like structure that catalyzes the majority of proteolysis in the cell [58]. It is made up of four axially stacked heptameric rings and in the case of the *T. acidophilum* proteasome that we study each of the rings consists of 7 equivalent subunits of either α or β polypeptides, arranged as α_7_β_7_β_7_α_7_. Initial studies by our laboratory focused on the α components of the proteasome for which we had obtained very nearly complete Ile, Leu, Val methyl assignments [24]. More recently we have focused on the β rings as these contain the catalytic residues for proteolysis, including Thr 1 whose nucleophilic hydroxyl moiety attacks a carbonyl carbon at the site of substrate cleavage [36]. Prior to this work we had prepared CP proteasome samples using commercially available U-[^13^C,^1^H]-Thr in a highly deuterated background. However, ^13^C,^1^H HMQC spectra, recorded with ^13^Cβ selective decoupling, were of poor quality and only a modest fraction of the expected cross peaks could be observed, Figure 5A. No doubt, the high level of protonation at the Thr β position contributes significantly to the poor quality of the data by increasing both transverse spin relaxation rates of methyl protons, leading to significant line broadening [10], and effective linewidths through the introduction of homonuclear scalar couplings. The added U-[^13^C,^1^H]-Thr results in labeling at the Ile δ1 position, as expected. The Ile correlations are also poor, reflecting both unresolved ^13^Cδ-^13^Cγ couplings and a single ^1^H at the Cγ position that derives from the protonated Thr. While improved Ile δ1 spectra could be obtained by addition of 2-ketobutyrate, it is not clear whether this precursor could completely turn off production of Ile biosynthesis from Thr.

With the development of a robust and economical biosynthesis scheme for the production of U-[^2^H],Thr-γ2[^13^CH_3_], optimally labeled for NMR studies of high molecular weight complexes, we have now revisited the NMR spectrum of the ^13^CH_3_-Thr labeled CP proteasome. To this end we have prepared a sample of U-[^2^H], Ile-δ1[^13^CH_3_], Thr-γ2[^13^CH_3_] α_7_β_7_β_7_α_7_, with labeling confined to the β rings. The HMQC data set is illustrated in Figure 5B and it is clear that the quality of the Thr region of the spectrum is much improved relative to when fully protonated precursor is used (compare Figures 5A and B). All 15 of the expected Thr correlations are now observed, although the intensities of a number of the cross peaks are low, indicating significant conformational heterogeneity that results from dynamics that are intrinsic to this allosteric protease.

Assignments for the majority of the Thr methyl groups could be obtained from NOE correlations (Supporting Information S1) connecting Thr peaks with those derived from the previously assigned Ile, Leu, Val methyls. Initially, a 2D ^13^C,^1^H edited NOESY data set was recorded (mixing time of 250 ms) on the U-[^2^H], Ile-δ1[^13^CH_3_], Thr-γ2[^13^CH_3_] α_7_β_7_β_7_α_7_ sample; analysis of the spectrum with the assistance of the X-ray structure of the *T. acidophilum* proteasome [59] lead to the assignment of 7 of the Thr methyls. Subsequently, analysis of 3D NOESY data recorded on a U-[^2^H], Ile-δ1[^13^CH_3_], Leu,Val-[pro*R*-^13^CH_3_,^12^CD_3_], Thr-γ2[^13^CH_3_] α_7_β_7_β_7_α_7_ sample confirmed the assignments, lead to the assignment of T81γ2 and T39γ2 and narrowed down the identities of a pair of peaks as belonging to either T106γ2 or T147γ2. Interestingly, assignments of 4 peaks derived from a cluster that includes Thr 1, 3,16 and 44 and that are weak have yet to be fully confirmed.

In summary, we have presented an enzymatic method for the synthesis of U-[^2^H], Thr-γ2[^13^CH_3_], optimized for NMR studies of high molecular weight proteins, starting from ^13^C-paraformaldehyde, natural abundance pyruvate and D_2_O. Metabolic enzymes from *E.coli* serve as catalysts. In total, 3 weeks are required to recombinantly produce and purify all five enzymes and to synthesize 1–2 grams of Thr. High quality spectra could be recorded on a 0.5 mM (subunit concentration) U-[^2^H], Ile-δ1[^13^CH_3_], Thr-γ2[^13^CH_3_] α_7_β_7_β_7_α_7_ sample and the quality of the data was far superior than in previous data sets obtained on samples generated with fully protonated Thr precursor. With the availability of U-[^2^H], Thr-γ2[^13^CH_3_] and previously described precursors it is now possible to label the methyl groups of any methyl containing amino acid. There are most certainly advantages for labeling each. The utility of Thr is made clear when one considers that, unlike other methyl containing residues, it has a high propensity for surface exposure [35] and it is more abundant at protein nucleic acid interfaces [34]. Moreover, Thr plays a critical role in the mechanisms of a number of important enzymes [36], [37] and in several eukaryotic signaling complexes where biological activity is regulated through phosphorylation [60]–[62]. The demonstration that high quality NMR spectra can be obtained of Thr methyl groups even for a complex of molecular mass approaching 700 kDa suggests that there will be a substantial number of systems that will benefit from the present labeling scheme.

## Materials and Methods

NMR spectra of compounds **2** and **4** are shown in Supporting Information S1 (**6** is shown in Figure 3) along with a detailed description of the biosynthesis of U-[^2^H],Thr-γ2[^13^CH_3_] and the expression and purification of the 5 enzymes (see Supporting Information S1) that are required for Thr production.

### Production of the Abp1p SH3 domain and NMR

Samples of U-[^13^C,^1^H] Abp1p SH3 domain were produced in *E.coli* BL21(DE3) cells, 0.3 L M9 minimal media per sample with ^13^C-glucose as the sole carbon source, using a protocol described in detail in Supporting Information S1. Samples ranged from 1.65–2 mM in protein, dissolved in 99.9% D_2_O, 50 mM NaH_2_PO_4_•H_2_O, 0.1 M NaCl, 1 mM NaN_3_, pH* = 7.0 buffer. ^12^C labeled precursors such as Thr, 2KB, Gly or 2-ketoisovalerate (2KIV) were added (depending on the sample) as indicated in Figure 4 and Supporting Information S1. The degree of precursor incorporation into protein has been assessed by quantifying cross peak intensities in constant-time ^13^C,^1^H HSQC data sets [63], [64] that were recorded of each sample and compared to a reference sample where precursors were not added. In order to account for slightly different protein concentrations in each sample the peaks of interest were normalized by the relative intensities of Ala methyl correlations. Ala methyl groups were chosen as an internal reference because they derive from pyruvate (glucose) and thus should not be affected by the addition of precursor.

### Proteasome production and analysis by NMR

Proteasome samples were prepared as described in detail in Supporting Information S1. A U-[^2^H], Ile-δ1[^13^CH_3_], Thr-γ2[^13^CH_3_] α_7_β_7_β_7_α_7_ sample (β subunit labeled) was generated by expression of the β subunit with 50 mg/L U-[^2^H], Thr-γ2[^13^CH_3_], 50 mg/L of labeled sodium 2-ketobutyrate (4-^13^C-3,3-d_2_) and 100 mg/L of d_5_-glycine, all added 1 hour before induction. A second sample, U-[^2^H], Ile-δ1[^13^CH_3_], Leu,Val-[pro*R*-^13^CH_3_,^12^CD_3_], Thr-γ2[^13^CH_3_] α_7_β_7_β_7_α_7_, was generated with 232 mg/L of an acetolactate based precursor added (purchased from Isotec, cat# 729558) in addition to 2-ketobutyrate. Samples were 0.54 (Ile) and 1.3 (Ile, Leu, Val) mM in protein concentration (subunit concentration), dissolved in 25 mM phosphate, 50 mM NaCl, 99.9% D_2_O buffer, pD = 6.8.

All NMR spectra were recorded at a static magnetic field of 18.8T, 70°C on a spectrometer equipped with a room temperature probe. Each ^13^C,^1^H HMQC data set was obtained in 2 hours. A 2D ^13^C,^1^H NOESY-HMQC data set was measured on the U-[^2^H], Ile-δ1[^13^CH_3_], Thr-γ2[^13^CH_3_] α_7_β_7_β_7_α_7_ sample with a 16.5 ppm spectral window in F_1_, 80 complex data points in the indirect dimension, 250 ms NOE mixing time and 1.5 s repetition delay for a total of 14.5 hours of accumulation. A 3D ^13^C,^13^C-separated NOESY experiment was recorded on the U-[^2^H], Ile-δ1[^13^CH_3_], Leu,Val-[pro*R*-^13^CH_3_,^12^CD_3_], Thr-γ2[^13^CH_3_] α_7_β_7_β_7_α_7_ sample, 18.5 ppm spectral window in both carbon dimensions, 8 scans per increment, 72 (t_1_) and 64 (t_2_) complex points in the two indirect dimensions, 200 ms NOE mixing time and 1.5 s interscan delay (75 hour experiment time).

## Supporting Information

Supporting Information S1 (DOC)
