# Supplementary material for: An Economical Method for Production of 2H,13CH3-Threonine for Solution NMR Studies of Large Protein Complexes: Application to the 670 kDa Proteasome
Source: PLoS One. 2012 Sep 11;7(9):e43725. doi: 10.1371/journal.pone.0043725 (PMC3439479; doi:10.1371/journal.pone.0043725)
Supplement: Supporting Information S1 — (DOC) [file pone.0043725.s001.doc]

Supporting Information

An Economical Method for Production of ^2^H,^13^CH_3_-Threonine for Solution NMR Studies of Large Protein Complexes: Application to the 670 kDa Proteasome

Algirdas Velyvis, Amy M. Ruschak and Lewis E. Kay

**Cloning of Enzymes**

Sequences of primers used for cloning of the 5 enzymes in Figure 2 with the unique portion of each primer that anneals to the gene encoding each enzyme underlined.

Table S1:

| Protein | Gene name(s) | 5' primer | 3' primer |
| --- | --- | --- | --- |
| KHGA | eda, hga, kdgA | cctgtattttcagggcgccatgaaaaactggaaaacaagt | ccgccaaaacagccaagcttttacagcttagcgccttct |
| BCAT | ilvE | cctgtattttcagggcgccatgaccacgaagaaagctg | ccgccaaaacagccaagcttttattgattaacttgatctaac |
| HSK | thrB | cctgtattttcagggcgccatggttaaagtttatgcccc | ccgccaaaacagccaagcttttagttttccagtactcgtg |
| TS | thrC | cctgtattttcagggcgccatgaaactctacaatctgaaa | ccgccaaaacagccaagcttttactgatgattcatcatcaat |
| AAT | aspC | cctgtattttcagggcgccatgtttgagaacattaccgc | ccgccaaaacagccaagcttttacagcactgccacaatc |

Template genomic DNA was prepared from 1 mL of a saturated culture of *E.coli* DH5α cells using an Easy-DNA kit (Invitrogen). The genes for each of the enzymes listed in Table S1 were PCR amplified from genomic DNA in reactions obtained by mixing 5 μL 10x Pfu buffer (containing Mg^2+^), 3 μL genomic DNA stock (approx. 100 ng/μL solution), 1 μL 10 mM dNTP mixture, 10 μL primer stock (contains 1 μM of 5' and 3' primers), 1 μL of Pfu DNA polymerase (2.5 units, Fermentas) and 30 μL H_2_O. PCR thermal cycling consisted of the following: 95 °C for 3 minutes, a block comprising the following 3 steps - 95 °C for 1 minute, 55 °C for 1 minute and 72 °C for 10 minutes - repeated 30 times followed by a final step of 72 °C for 10 minutes. Product DNA was purified with a QIAquick PCR purification kit (Qiagen), resulting in 50-70 ng/μL of product.

Amplified genes were inserted into a pProEX Htb vector by means of “restriction free” cloning, conceptually similar to QuickChange point-mutagenesis [1,2]. The common part of each primer (not underlined) in Table S1 anneals to a region in the MCS of pProEX Htb so that the PCR products from the previous step (above) can be considered as megaprimers with the top and bottom strands serving as primers for amplification of the vector containing the gene of interest. Reaction mixtures were prepared by mixing 5 μL 10x Pfu buffer, 3 μL of 10 ng/μL pProEX Htb, 1 μL 10 mM dNTP mixture, 10 μL megaprimer (containing entire gene to be cloned), 1 μL of Pfu Turbo (2.5 units, Agilent) and 30 μL H_2_O. Thermal cycling consisted of the following steps: 95 °C for 2 minutes, a block comprising a cycle of 95 °C for 0.5 minute, 55 °C for 1 minute and 68 °C for 15 minutes - repeated 35 times and a final step of 68 °C for 15 minutes. Subsequently, 1 μL of DpnI (20 units; NEB) was added to degrade parental DNA and reactions incubated for 4.5 hours at 37 °C. 3 μL of cloning reactions of KHGA, HSK and TS were electroporated into *E.coli* directly, since by agarose gel electrophoresis (5 μL of reaction mixture) a high concentration of reaction product was detected for these proteins. For BCAT and AAT the concentration of product was much lower. All of the DNA produced was therefore concentrated by ethanol precipitation and resuspended into 5 μL of water of which 3 μL were electroporated into *E.coli*. Positive clones were isolated for all 5 target enzymes and the correct DNA sequence confirmed by sequencing. In each case the expressed protein contains an N-terminal hexahistidine tag, followed by a TEV protease cleavage site and the full length target enzyme sequence.

**Enzyme expression and purification**

All proteins were expressed in BL21(DE3) *E.coli* cells. Saturated overnight cultures were used to inoculate LB medium. For all proteins except TS, 1 L of culture was grown at 37 °C, 300 rpm shaking, 1 mM of IPTG added at OD_600_ 0.75-0.95 and growth continued for 4-5 hours under the same conditions. For TS, 2 L of LB were supplemented with 180 g/L of D-sorbitol and 2.5 mM betaine [3], cultures grown at 37 °C and 300 rpm shaking, 1 mM IPTG added when OD_600_ reached 0.8, temperature lowered to 18 °C and culture harvested 20 hours later. Cultures of PLP-dependent enzymes (TS, BCAT and AAT) were also supplemented with 10 mg/L of PLP (Sigma P-9255) at the time of induction. After harvesting, cell pastes were resuspended in 20-30 mL of buffer for Ni chromatography (50 mM NaH_2_PO_4_, 0.2 M NaCl, 10 mM imidazole, 1.5 mM NaN_3_, pH=8) and frozen.

Upon purification cell suspensions were thawed, treated with 1-2 mg/mL lysozyme for 15 minutes at room temperature, supplemented with 2-4 mg (total) of DNaseI and 1 tablet of EDTA-free “Complete” protease inhibitor cocktail (for enzymes containing PLP cofactor, the suspension was additionally supplemented with 10 mg of PLP) and sonicated on ice to disrupt the cells. Lysates were cleared by centrifugation for 30 minutes at 40000x g followed by filtration through a 0.8 μm filter. A 5 mL HisTrap column was equilibrated with lysis buffer, cleared lysates applied to the column at room temperature and the resin washed with 10 volumes of lysis buffer. Proteins were eluted in 10-20 mL of buffer containing 0.1 M NaH_2_PO_4_, 0.3 M imidazole, 1.5 mM NaN_3_ pH=7.8. PLP-dependent enzyme preparations were supplemented with 0.5 mM PLP, all proteins except HSK were mixed with an equal volume of glycerol and stored at -20 °C. HSK eluted from the HisTrap column was dialyzed against 2 L of 50 mM NaH_2_PO_4_, 0.1 M NaCl, 20 mM beta-mercaptoethanol, pH=7.4 buffer overnight at 5 °C. Dialyzed HSK was supplemented with one tablet of EDTA-free “Complete” protease inhibitor cocktail and with ~10 mM EDTA, mixed with an equal volume of glycerol and stored at -20 °C. Protein concentrations were determined by the Bradford assay and found to be higher by 20-30% compared to estimates based on measurement of A_280_ and absorption coefficients computed by adding contributions from Tyr, Trp, and PLP.

**Thr Biosynthesis**

All reagents and buffer components are natural abundance unless indicated otherwise. Compounds referred to as **1**-**6** are as indicated in Figure 2.

Preparation of **1b**: 1 day before the start of reaction 1 (see Figure 2), overnight dialysis of 8 mL of KHGA stock in 50% glycerol (15 mg total enzyme, 0.6 μmol) was carried out against 4L of 50 mM imidazole, 0.1 M NaCl, pH=7.5 buffer at 5 °C. Next day, dialyzed KHGA was concentrated to <1 mL and exchanged into 50 mL of 50 mM imidazole, 0.1 M NaCl pH*=7.4, 99.9% D_2_O buffer by diluting with 10 mL of D_2_O buffer and concentrating to <1 mL three times. Final volume of catalyst was adjusted to 1 mL in D_2_O buffer. 122 mg of imidazole and 902 mg (8.2 mmol, 2 equivalents with respect to formaldehyde, see step below) of sodium pyruvate (Sigma P8574) were dissolved in 25 mL 99.9% D_2_O, pH* was adjusted to 7.5 with DCl in D_2_O. KHGA solution was added to pyruvate and incubated at room temperature for 2 hours to complete H/D exchange.

Preparation of **2**: 127 mg (4.1 mmol) of ^13^C-paraformaldehyde (CIL CLM-229-1), 50 mg of Na_2_HPO_4_ and 1 μL of 40% NaOD in D_2_O were added to 5.2 mL of 99.9% D_2_O. Suspension was incubated at 60 °C until all paraformaldehyde dissolved (approximately 30 minutes, some amount of fluffy gray precipitate appeared towards the end of the incubation). Solution was cooled down to room temperature and pH* adjusted to 7.5 with DCl in D_2_O. Depolymerized ^13^C-formaldehyde was added into pyruvate and KHGA solution, volume adjusted to 36 mL with D_2_O, resulting in a solution containing 50 mM imidazole, 0.11 M formaldehyde and 0.22 M pyruvate; pH* was found to be 7.15 and was adjusted to 7.2 with NaOD in D_2_O. Reaction 1 was incubated at room temperature overnight with essentially complete conversion of formaldehyde into **2** by the next day as established by NMR. The reaction mixture was filtered through a concentrator unit to remove KHGA (35 mL of filtrate and 0.5 mL of concentrate containing enzyme); the concentrator was additionally washed with 0.5 mL of Tris/D_2_O buffer and ~0.5 mL of “wash” was added to the first 35 mL of filtrate.

Preparation of **4** (reaction 2-3): 3 days before reaction, 3.393 g (6.15 mmol, 1.5 equiv.) of disodium ATP (Sigma A3377), 1.152 g (6.15 mmol, 1.5 equiv) of sodium glutamate monohydrate (Sigma G-5917), 80.8 mg of MgSO_4_•7H_2_O, 226 mg of Tris and 147 mg of NaOH were dissolved in 99.9% D_2_O (20 mL) and freeze-dried to remove exchangeable hydrogens. One day before reaction 2-3, dialysis of 7 mL of AAT stock in 50% glycerol (46 mg of protein, 1 μmol) and 18 mL of HSK stock in 50% glycerol (41 mg of protein, 1.1 μmol) was carried out against 4 L of 50 mM TrisHCl, 0.1 M NaCl, pH=8 buffer, 5 °C. The next day 100 mL of 50 mM TrisHCl, 0.1 M NaCl, pH*=8, 99.9% D_2_O was prepared; dialyzed AAT and HSK were concentrated to <1 and 2 mL respectively in concentrators, and exchanged into the Tris/D_2_O buffer by diluting with 10 mL of buffer and concentrating to 1 mL three times. Final volumes were 1 mL of AAT and 2.25 mL of HSK. Freeze-dried mixture of ATP, glutamate, MgSO_4_ and Tris was dissolved in filtrate of reaction 1 (containing product **2**), and 9.9 mg of PLP (Sigma P-9255) was added; pH* was found to be 6 and was adjusted to 8.0 with NaOD in D_2_O. AAT and HSK solutions were added to obtain 42.5 mL of reaction 2-3. In addition to imidazole and excess of pyruvate **1b** carried over from reaction 1, the solution contains ~50 mM Tris buffer, ~0.1 M of **2**, ~0.15 M of glutamate, ~0.15 M of ATP, ~1 mM of PLP and ~8 mM of Mg^2+^. The reaction mixture was incubated under aluminum foil at room temperature for up to 40 hours. Note that the process of phosphorylation to produce **4** leads to acidification of the reaction solution and ultimately to a decrease in enzyme activity. Thus, pH* was adjusted manually every 12 -20 hours. For example, pH* was found to drop to 7.2 in the first 16.5 hours, during which 47% of **2** had converted to **4** based on NMR analysis. pH* was adjusted to 8.3 with NaOD in D_2_O and DCl in D_2_O and found to be 7.95 24.5 hours after beginning of the incubation and 7.8 at 40.5 hours, when 80% conversion to **4** was observed. The reaction mixture was filtered through a concentrator to obtain 39 mL of filtrate; catalyst-containing concentrate was further washed with 4 mL of D_2_O and the “wash” fraction was combined with the main volume of filtrate and stored at 4 °C until purification.

Purification of **4**: D_2_O solution containing **4** was diluted with 370 mL of H_2_O (10-fold dilution) and subsequently purified by chromatography using ~35 mL of Q-sepharose FF anion exchanger resin packed into a 16 mm diameter column. The solvent system used for product elution was a mixture of solvent A, milliQ water, and solvent B, 0.5 M NaCl in H_2_O. Chromatography consisted of washing the resin (Cl^-^ form) in water, loading 20 mL of diluted reaction 2-3, washing with water, eluting with a 0-30% gradient of B in 100 mL and washing with 100% solvent B. Fractions containing **4** (150-168 mL) elute at ~100 mM NaCl. While there is no UV absorbance at 280 nm for **4**, its elution can be detected by a small dip in solution conductivity. To determine the precise location of the elution peak of **4**, 2 mL fractions were collected from the 1^st^ 20 mL injection and analyzed by ninhydrin for amino acid content (which also reveals strong amino reactivity in the flow-through and early NaCl gradient fractions corresponding to glutamate, as determined by NMR analysis). Fractions containing the entire purified **4** (~400 mL) were pooled and solvent removed on a rotary evaporator to near dryness (~5 mL final volume).

Preparation of **5**: A day before reaction, dialysis of 8 mL of TS stock in 50% glycerol (48 mg of protein, 0.96 μmol) was carried out against 5 L of 50 mM NaH_2_PO_4_, 0.1 M NaCl pH=7.5 buffer for 6 hours and then against 4 L of freshly made buffer of the same composition overnight at 5 °C. After dialysis, TS was concentrated to 4 mL. Solution of **4** containing slurry of saturated NaCl was diluted to 24 mL, 3.7 mg of PLP added (intended concentration is 0.5 mM in 30 mL), pH adjusted to 6.4-6.5 followed by addition of the solution of TS and the volume adjusted to 30 mL. The reaction was incubated at room temperature for 2 hours, with complete conversion of **4** into **5**, as established by NMR. At this point the reaction mixture was filtered through a concentrator to remove catalyst, concentrate (1 mL) additionally washed with 2 mL of H_2_O and the “wash” fraction combined with the rest of **5**.

Preparation of **6**: A day before the reaction dialysis of 17 mL of BCAT stock in 50% glycerol (74 mg, 2.0 μmol) was carried out overnight, 5^o^C, against 4 L of 50 mM NaH_2_PO_4_, 0.1 M NaCl buffer, pH=7.5. The following day 50 mL of 50 mM Na_2_HPO_4_, 0.1 M NaCl, pH*=7.5 buffer, 99.9% D_2_O was prepared, dialyzed BCAT concentrated to <1 mL and exchanged into D_2_O buffer by dilution to 10 mL, concentrated to 0.5 mL in a process that is repeated three times (final volume of enzyme solution was 1.5 mL). Water was removed to dryness from the solution of **5** by means of rotary evaporation. The dry material, consisting of **5**, a nearly equimolar amount of phosphate, about 2.1 g of NaCl and 3.7 mg of PLP was dissolved in 27.5 mL of 99.9% D_2_O; pH* was 7.25 and left as is. A reference ^1^H NMR spectrum was recorded, the solution of BCAT added, the total volume adjusted to 30 mL and the reaction was incubated for 20 hours at 37 °C under aluminum foil. The height of the residual Hα proton signal in the ^1^H NMR spectrum subsequently recorded is about four times that of the ^13^C-satellite of the same line prior to H/D exchange, implying 98% conversion of **5** to **6**. The reaction mixture was passed through a concentrator to remove catalyst and the concentrate additionally extracted with 3 mL of D_2_O to yield 32 mL of product solution.

A calibration curve for ninhydrin was constructed using a stock of unlabeled Thr and used to measure the concentration of **6** in the final D_2_O solution, which was found to be 77.7 mM (2.49 mmol, 304 mg, 61% yield). No attempt was made to account for the fact that NMR spectra show several ^13^C-labeled impurities, the most prominent three with peak heights 0.5-1% of the corresponding peak height of **6**. Most likely these impurities are also amino acids such as L-allo-threonine and D-allo-threonine, so that the ninhydrin assay may slightly overestimate the concentration of **6**. The solution of **6** was stored in aliquots containing 50 mg of the compound at -80 °C. No changes in ^1^H NMR spectrum were detected during 3 months of storage.

Additional notes relevant to the synthesis of **6**

(1) In a preliminary trial we found that if AAT and HSK are added to reaction 2-3 in H_2_O buffers (adding about 10% of H_2_O into the filtered D_2_O solution of **2**), up to 7% ^1^H was introduced into the formerly perdeuterated C3 position. This likely reflects the acidity of the C3 position adjacent to the keto group in **2** and extended incubation (40 hours) that is required for reaction 2-3 to go to near completion. To prevent introduction of ^1^H into **2** it is necessary to perform reaction 2-3 in a solution with as high a D_2_O content as possible. To this end we exchanged both AAT and HSK enzymes into D_2_O-based buffers prior to adding them into the reaction. We also exchanged buffer components and reactants glutamate and ATP into deuterated forms (labile positions) by freeze-drying from D_2_O.

(2) We found that using Tris to buffer reaction 1 caused the appearance of unknown impurities with ^1^H NMR signals in the 3.5-4.5 ppm region. Thus imidazole was chosen to buffer the reaction.

(3) We have stated in the manuscript that the hydrogen added by TS to the C4 position in the conversion of **4** to **5** derives from solvent. However, the catalytic mechanism of TS is very complex [4] and while the oxygen atom must come from water the origin of the hydrogen atom is, in fact, less clear. Interestingly, in the TS-catalyzed conversion of fully protonated OPHS in solutions containing a small fraction (6-12%) of H_2_O in D_2_O we have observed formation of the CH_3_ isotopomer of Thr in an amount that is approximately 5-fold higher than what would be predicted based on the fraction of H_2_O present. This implies either a very strong kinetic isotope effect (a distinct possibility) or possibly that some fraction of the hydrogen atoms abstracted by the enzyme from the C3 position of OPHS (a vinyl intermediate is formed) do not exchange with the solvent and hence can be transferred to the C4 position – the nascent methyl group. However, our sample of **6** shows no evidence of the CH_2_D isotopomer that would be expected if this mechanism were operative, implying that under the conditions of our reactions the additional hydrogen at C4 does indeed derive from solvent.

(4) We store all catalysts at -20 °C in glycerol to prevent freezing. While standard in biochemistry laboratories it is inconvenient as volumes of dialyzed solutions start exceeding 50 - 100 mL. Lyophilization of enzymes is an alternative solution for intermediate to long term storage and it alleviates the need for D_2_O buffer exchange. However, we have not established whether lyophilization negatively impacts the catalytic activity of any of the enzymes that we have used in the synthesis.

**Abp1p SH3 domain expression and purification**

Plasmid “Abp TRX” [5], which encodes a fusion protein consisting of thioredoxin, hexahistidine tag, TEV protease cleavage site and an SH3 domain of the yeast protein Abp1p, was transformed into *E.coli* BL21(DE3) and saturated overnight cultures were grown in LB medium. M9 minimal medium in H_2_O was prepared with 0.5 g/L of ^15^N NH_4_Cl and 3 g/L of ^13^C_6_-glucose, seeded with cells harvested from an overnight culture and grown at 37 °C with 250 rpm shaking. One hour before projected induction time (optimal OD_600_ for induction is 0.8) 0.3 L cultures were aliquoted and supplements were added where needed (see Table S2, below). IPTG and Thr in some cultures were added 1 hour later and the temperature lowered to 20 °C. Cultures were harvested 16-20 hours after induction and stored frozen in lysis buffer (50 mM NaH_2_PO_4_, 6 M Guanidine hydrochloride, 5 mM imidazole, pH=7.7). The following supplements were used, with amounts and addition times specified in Table S2: natural isotopic abundance L-Threonine (Sigma T-8534), natural abundance sodium 2-ketobutyrate (2KB; Sigma K-0875), natural abundance sodium 2-ketoisovalerate (2KIV; Sigma K-0128) and ^13^C_2_-glycine (CIL # CLM-1017).

Table S2:

| Culture # | L-Thr | 2KB, 1 hour before induction | 2KIV, 1 hour before induction | ^13^C-glycine, 1 hour before induction |
| --- | --- | --- | --- | --- |
| 1 | - | - | - | - |
| 2 | - | - | - | - |
| 3 | 50 mg/L at induction time | - | - | - |
| 4 | 50 mg/L at induction time | 50 mg/L | - | - |
| 5 | 50 mg/L at induction time | 50 mg/L | 80 mg/L | - |
| 6 | 100 mg/L at induction time | - | - | - |
| 7 | - | - | - | 300 mg/L |
| 8 | 50 mg/L 1 hour before induction | 50 mg/L | - | 100 mg/L |
| 9 | 50 mg/L 1 hour before induction | 50 mg/L | - | 300 mg/L |
| 10 | 100 mg/L 1 hour before induction | - | - | 100 mg/L |
| 11 | 100 mg/L 1 hour before induction | - | - | 300 mg/L |

To purify proteins (0.3 L cell culture per NMR sample), cell suspensions were sonicated to disrupt the cells and lysates cleared by centifugation (20 minutes at 40000x g) followed by filtration through a 0.8 μm filter. A 1 mL HisTrap column was equilibrated with lysis buffer, cleared lysates applied to the column at room temperature and the resin washed with 15 volumes of lysis buffer. Proteins were eluted in 5 mL of high imidazole buffer (50 mM NaH_2_PO_4_, 6 M Guanidine hydrochloride, 0.3 M imidazole, pH=8). For refolding, each protein preparation was diluted to ~ 1g/L protein concentration with lysis or elution buffer, supplemented with 1 mM DTT and dialyzed against 1.5-2.5 L of 50 mM Tris-HCl, 0.1 M NaCl, 1 mM EDTA, 2 mM DTT buffer overnight at 5 °C. The following day 2 mg of TEV protease were added into each dialysis bag and dialysis continued for another day at room temperature against a fresh 2-2.5 L volume of identical buffer. After TEV cleavage, each sample was filtered through a 0.4 μm filter, supplemented with 10 mM imidazole and passed through a 5 mL HisTrap column to remove thioredoxin, TEV protease and any uncleaved thioredoxin-SH3 fusion proteins. Flow-through fractions containing SH3 domain samples were retained for the next step (the presence of 1 mM EDTA in the dialysis buffer causes a slow drainage of Ni^2+^ from the HisTrap resin; up to two cleaved SH3 samples can be processed with a single 5 mL HisTrap column before the resin requires recharging with Ni^2+^). Protein samples were further purified by means of a MonoQ column (8 mL bed volume). The resin was equilibrated in 50 mM NaH_2_PO_4_, pH=6.8, the entire SH3 domain sample loaded and eluted with a 0-100% gradient of 50 mM NaH_2_PO_4_, 1 M NaCl, pH=6.8 buffer in 50 mL. SH3-containing fractions (eluting at ~0.5 M NaCl) were pooled, concentrated to <0.5 mL and exchanged into D_2_O NMR buffer (99.9% D_2_O, 50 mM NaH_2_PO_4_•H_2_O, 0.1 M NaCl, 1 mM NaN_3_, pH*=7.0) by dilution with 10 mL of buffer and concentration to < 0.5 mL twice. Final volume of each NMR sample was adjusted to 505-510 μL; SH3 domain concentration was found from the A_280_ value of protein diluted in 8 M Guanidine hydrochloride, assuming ε_280_=20970 M^-1^cm^-1^, and ranged from 1.7 to 2.0 mM.

**Proteasome preparation**

Both U-[^2^H], Ile-δ1[^13^CH_3_], Thr-γ2[^13^CH_3_] α_7_β_7_β_7_α_7_ and U-[^2^H], Ile-δ1[^13^CH_3_], Leu,Val-[pro*R-*^13^CH_3_,^12^CD_3_], Thr-γ2[^13^CH_3_] α_7_β_7_β_7_α_7_ samples (β subunit labeled in both cases) were generated, as described below. The precursor for the generation of the pro*R* ^13^CH_3_ labeling of Leu,Val methyl groups is purchased as an ester that must be saponified prior to use as described: 420 μL of neat ethyl 2-hydroxy-2-methyl-d_3_-3-oxo-butanoate-4-^13^C (approx. 464 mg; this amount of material is intended for 2 L of culture and corresponds to 100 mg/L of 2-ketoisovalerate) is mixed with 2 mL of 9 g/L Na_2_HPO_4_ (incomplete dissolution). 10 N NaOH was added in 3 increments of 100 μL each, with the pH in the 12.4-12.6 range for 5 minutes after the 3rd increment, indicating that saponification is complete. An additional 1 mL of 9 g/L Na_2_HPO_4_ was added to achieve better dissolution. Completeness of ester hydrolysis was confirmed by ^1^H NMR; 200 μL of approximately 1 M HCl was added to adjust the pH to 7 and the precursor stored at 4 °C.

To produce proteins, plasmids “αS95C” (encoding a hexahistidine tag, TEV protease cleavage site and an S95C mutant of the α-subunit of *T. acidophilum* proteasome) and “βWT” (encoding a fusion protein of NusA, hexahistidine tag, TEV protease cleavage site and wild-type β-subunit of *T. acidophilum* proteasome) were transformed into BL21(DE3) Codon Plus (RIL) cells. Starter cultures containing kanamycin and chloramphenicol were grown at room temperature with shaking until OD_600_ reached 0.9-1, at which point cells were harvested and used to seed D_2_O-based M9 cultures. M9 media was prepared in 99.9% D_2_O with 3 g/L of d_7_-glucose and contains kanamycin only (no chloramphenicol). For expression of α-subunit, 0.5 L of D_2_O-M9 was seeded to an OD_600_=0.02 and cells were grown at 37 °C and 180 rpm overnight; subsequently shaking increased to 300 rpm, culture grown to OD_600_=0.9, induced with 1 mM IPTG and growth continued under the same conditions. Cells were harvested 20 hours later and stored frozen in Ni lysis buffer (50 mM NaH_2_PO_4_, 0.5 M NaCl, 30 mM imidazole, 1.5 mM NaN_3_, pH=8). For expression of the β-subunit, 3 L of D_2_O-M9 were seeded to an OD_600_=0.1 in flasks containing not more that 0.5 L of medium and grown at 37 °C and 300 rpm. Upon reaching OD_600_ 0.7-0.75, additives were added. To 1 L of culture 50 mg of synthesized labeled Thr **6**, 50 mg of sodium 2-ketobutyrate (methyl-^13^C-3,3-D_2_; CIL # CDLM-7318-0.5) and 100 mg of glycine-d_5_ (CIL # DLM-280-5) were added. The remaining 2 L of culture were supplemented with 100 mg of synthesized labeled Thr **6**, 100 mg of labeled sodium 2-ketobutyrate, 200 mg of glycine-d_5_ and the entire volume of processed Leu,Val-[*R*-^13^CH_3_,^12^CD_3_] precursor (see above). The temperature was dropped to 18 °C, IPTG (1mM) was added 1 hour later (OD_600_ 0.9-1) and protein production was allowed to proceed for 17 hours. Harvested cell paste was stored in Ni lysis buffer (50 mM NaH_2_PO_4_, 0.2 M NaCl, 10 mM imidazole, 1.5 mM NaN_3_, pH=8), frozen.

The suspension of cells expressing the α-subunit was thawed, supplemented with 15 mg of lysozyme and incubated at room temperature for 15 minutes. Cells were disrupted by sonication and 1 tablet of EDTA-free “Complete” protease inhibitor cocktail was added. Lysate was cleared by centrifugation for 20 minutes at 40000x g and filtered through a 0.8 μm filter. A 5 mL HisTrap HP column was equilibrated with lysis buffer, cleared lysate applied to the column at room temperature and the resin washed with 8 volumes of lysis buffer. Elution in 25 mL of buffer containing 0.1 M NaH_2_PO_4_, 0.3 M imidazole, 1.5 mM NaN_3_ pH=7.8 afforded ~82 mg of protein. 8 mg of TEV protease were added to the purified α-subunit and protein was dialyzed against 4 L of 50 mM Tris-HCl, 1 mM EDTA, 2 mM DTT buffer overnight at 5 °C. After TEV cleavage, protein was concentrated and purified on a Superdex 200 gel filtration column (120 mL bed volume) at room temperature, with 50 mM NaH_2_PO_4_ 0.1 M NaCl pH=7.5 buffer flowing at 1 mL/minute. Fractions containing the α-subunit were stored at 4 °C until reconstitution of the proteasome holoenzyme.

Suspensions of cells expressing the β-subunit were thawed, supplemented with 12 mg of lysozyme per 1 L of cell culture and incubated at room temperature for 20 minutes. Cells were disrupted by sonication and lysates were cleared by centrifugation for 30 minutes at 40000x g and filtered through a 0.8 μm filter (lysate from 1 L of culture was filtered as is; lysate from 2 L of culture was supplemented with 0.3 mg of DNaseI (Roche) a few minutes before filtration). A 5 mL HisTrap HP column was equilibrated with lysis buffer, cleared lysate applied to the column at room temperature and the resin washed with 8 volumes of lysis buffer. Proteins were eluted in 15-20 mL of buffer containing 0.1 M NaH_2_PO_4_, 0.3 M imidazole, 1.5 mM NaN_3_, pH=7.8. Since SDS-PAGE indicated that the Ni flow-through fraction has a significant amount of fusion protein in the case of the 2 L culture, this fraction was purified over the HisTrap column again, resulting in an additional ~20% NusA-β-subunit fusion protein. 2-3 mg of TEV protease and 5 mM DTT were added to purified proteins that were then subsequently dialyzed against 4 L of 25 mM Tris-HCl, 1 mM EDTA, 2 mM DTT buffer overnight at 5 °C. After TEV cleavage, proteins were purified on a MonoQ, 8 mL column: the resin was equilibrated in 50 mM Tris-HCl, pH=8.0, the entire protein volume loaded and eluted with a 0-60% gradient of 50 mM Tris-HCl, 1 M NaCl buffer, pH=8.0, in 60 mL. Fractions containing β-subunit (eluting at ~0.2 M NaCl) were pooled.

To reconstitute the proteasome holoenzyme, the entire volume of a given β-subunit preparation was mixed with 20% excess of α-subunit (concentrations determined spectrophotometrically assuming ε_280_ values of 16390 and 13410 M^-1^cm^-1^ for α- and  β-subunits respectively), incubated at 37 °C for 6 hours, concentrated to 0.6 mL and further incubated at 37 °C overnight. The following day the holoenzyme preparations were purified on a Superdex 200 gel filtration column (120 mL bed volume) at room temperature, with 50 mM NaH_2_PO_4_, 0.1 M NaCl, pH=7.5 buffer flowing at 0.7 mL/minute. Fractions containing holoenzyme were pooled and exchanged into NMR sample buffer. 50 mL of 10x stock of D_2_O NMR buffer were prepared by dissolving 0.910 g of KH_2_PO_4_, 1.01 g of K_2_HPO_4_, 1.462 g of NaCl , 0.150 g of NaN_3_ and 0.186 g of EDTA in 50 mL of 99.9% D_2_O. The proteasome holoenzyme was concentrated to < 0.5 mL and exchanged into 1x D_2_O buffer (25 mM phosphate and 50 mM NaCl) by dilution - concentration twice. Final volumes of NMR samples were adjusted to 450 μL; concentrations were determined from A_280_ values of protein diluted in 8 M Guanidine hydrochloride and were 0.536 mM and 1.285 mM for U-[^2^H], Ile-δ1[^13^CH_3_], Thr-γ2[^13^CH_3_] α_7_β_7_β_7_α_7_ (1 L of culture) and U-[^2^H], Ile-δ1[^13^CH_3_], Leu,Val-[pro*R-*^13^CH_3_,^12^CD_3_], Thr-γ2[^13^CH_3_] α_7_β_7_β_7_α_7_ (2 L of culture) samples, respectively.


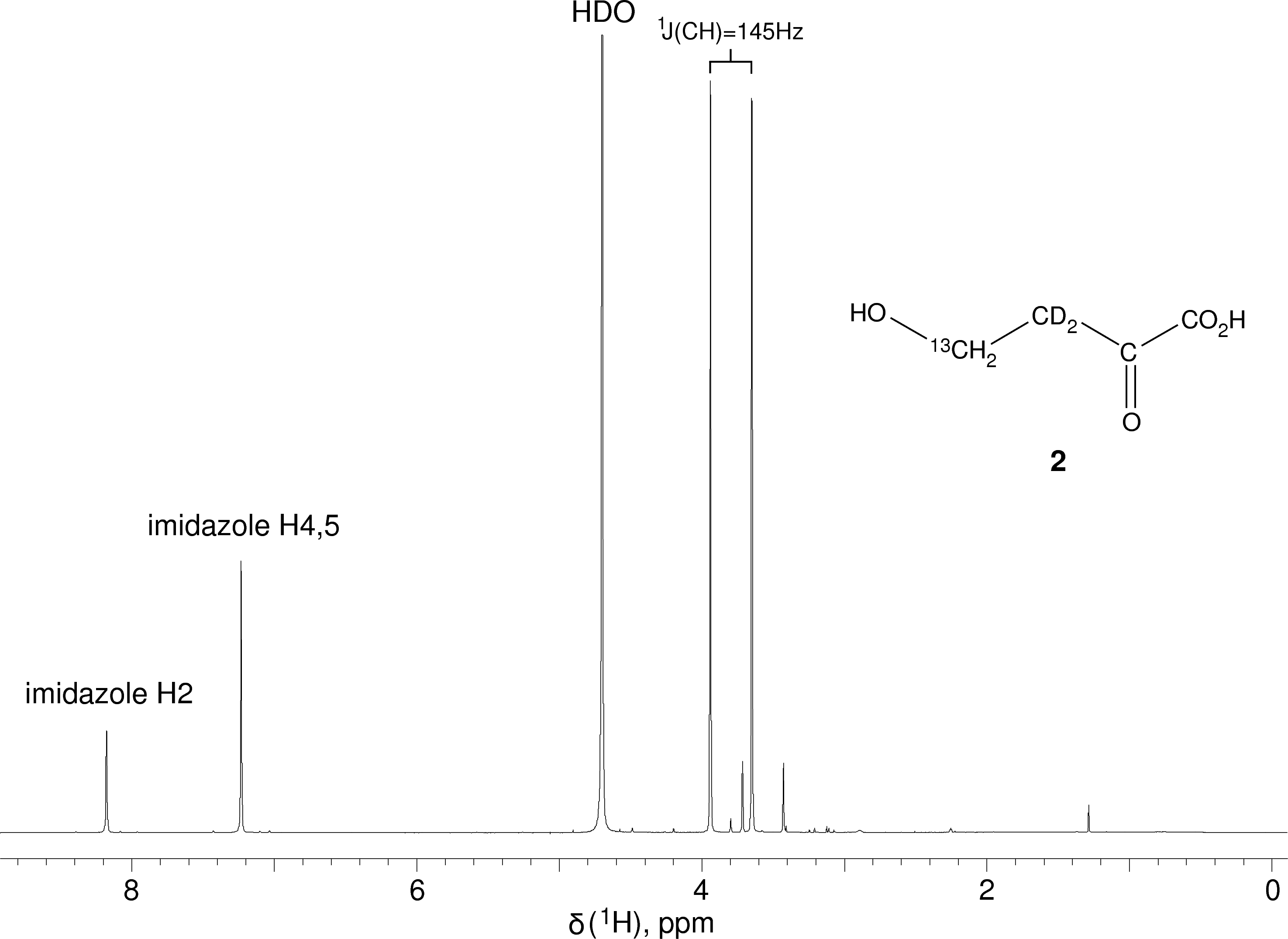


Figure S1. ^1^H NMR spectrum of reaction 1 upon completion. Only product **2** is present (see Figure 2). Protons from the reactant ^13^CH_2_O resonate at 4.6 and 4.95 ppm. The absence of such peaks indicates that the reaction has gone to completion.


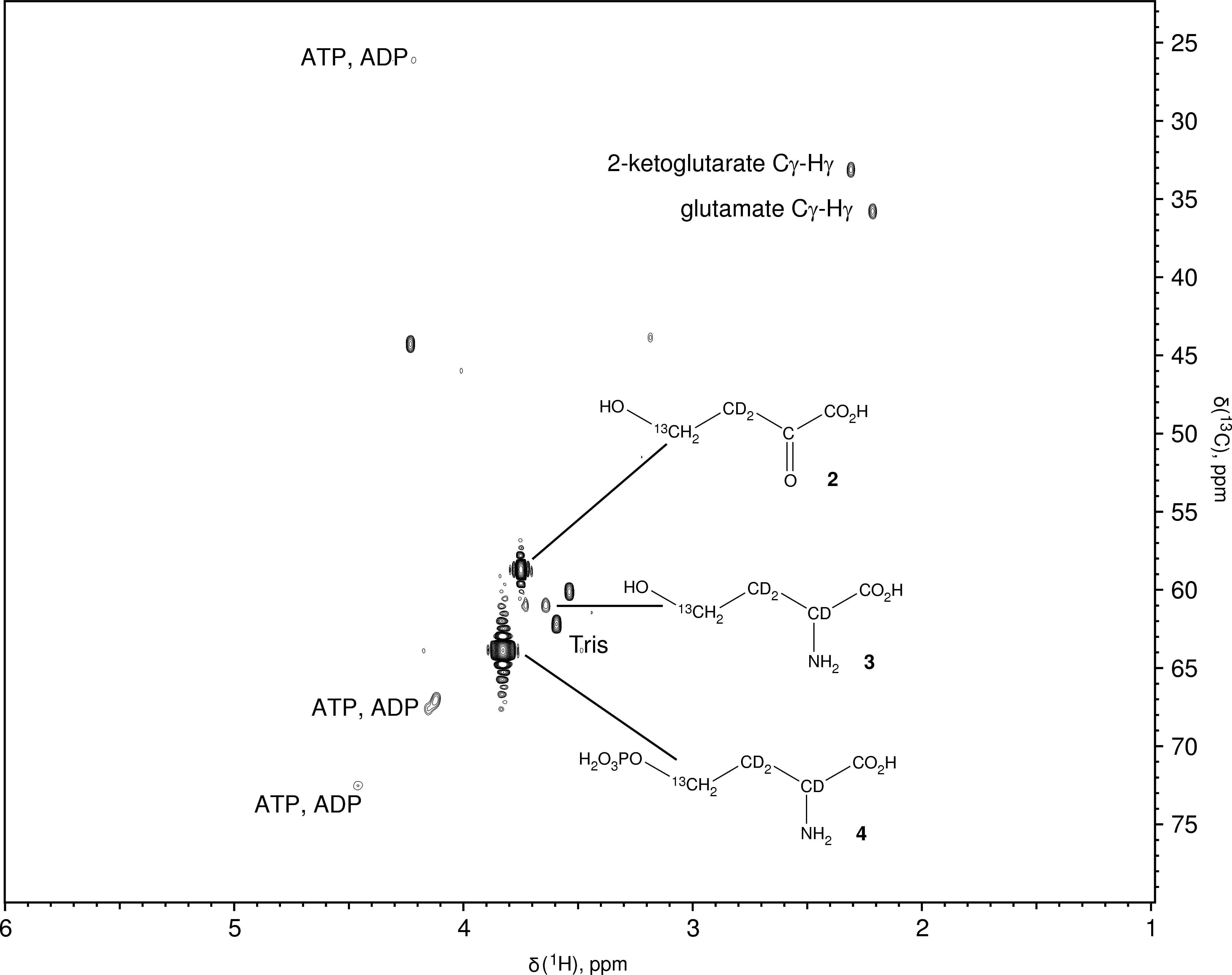


Figure S2. ^13^C,^1^H HSQC spectrum of reaction ‘2-3’ after 40 hours showing that the expected product O-phospho-L-homoserine, **4**, is much more abundant than the intermediate homoserine (80%:1%), with 20% starting material, **2**, remaining.


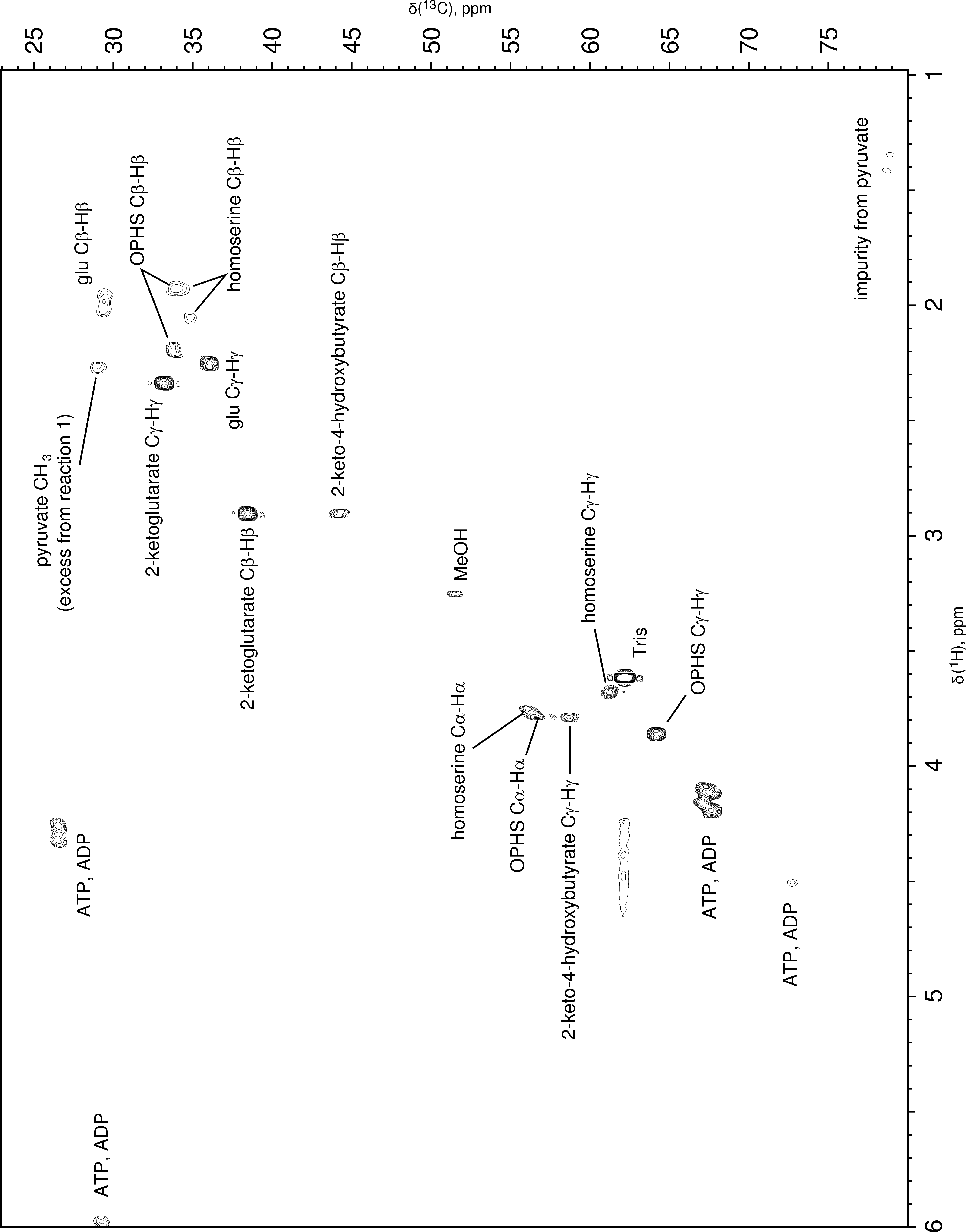


Figure S3. Annotated ^13^C,^1^H HSQC spectrum of reaction ‘2-3’ at an intermediate stage of the reaction performed in ^1^H_2_O solvent with all reagents protonated and of natural isotopic abundance. At this stage homoserine is present but after 40 hours it becomes less than 1% of the product OPHS (Figure S2). Reaction mixture was diluted 1:9 with D_2_O for the NMR sample. OPHS = O-phospho-L-homoserine (non-isotopically enriched version of **4**).


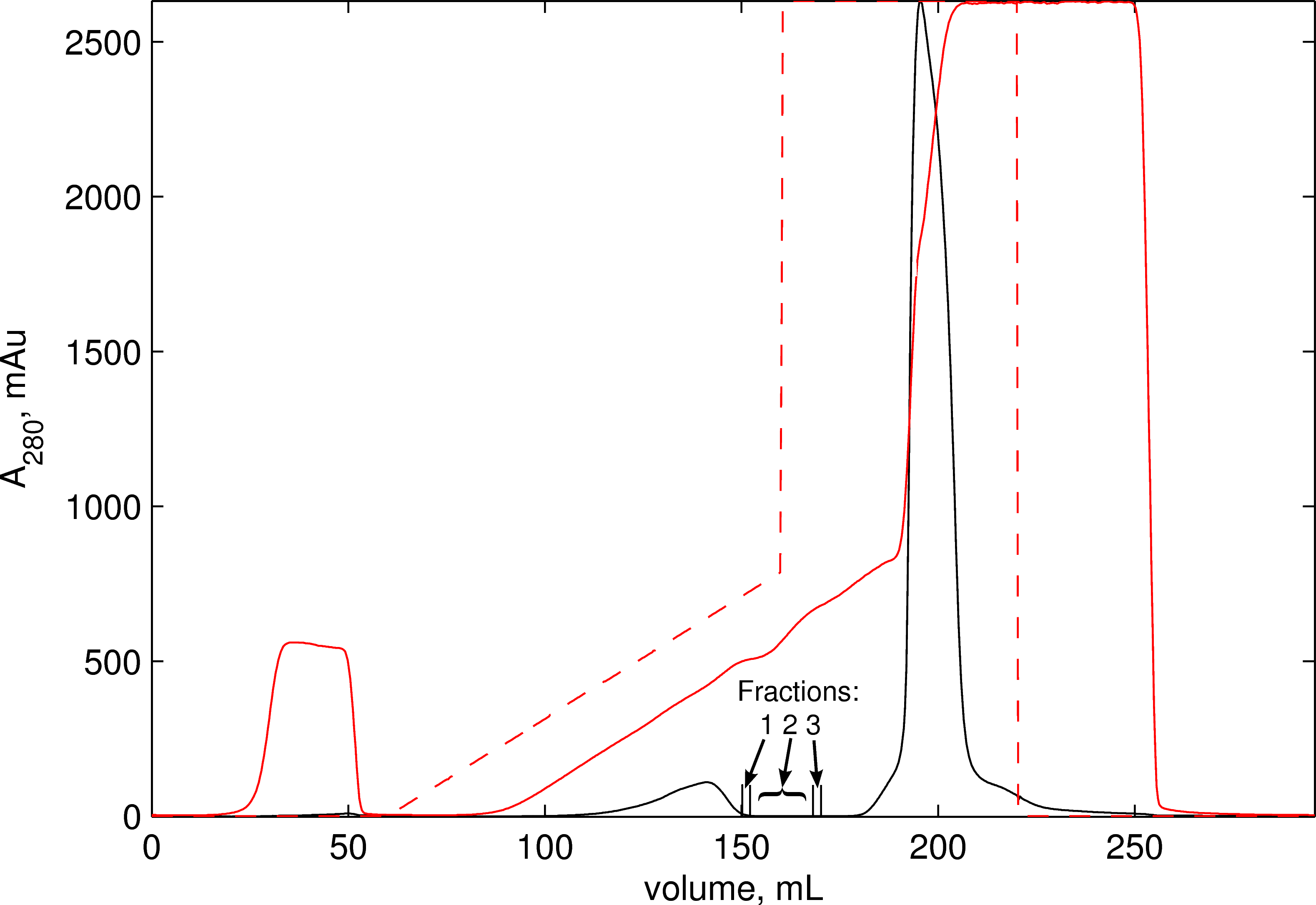


Figure S4. Purification of **4** on a Q-sepharose FF column (quaternary ammonium-based anion exchanger resin), as described in text. Black line is A_280_, red line conductivity and dashed red line denotes the solvent gradient at the pump. Collected fractions are indicated. Fraction 3 contains only a negligible concentration of **4** and was discarded while fractions 1 and 2, containing **4**, were pooled.


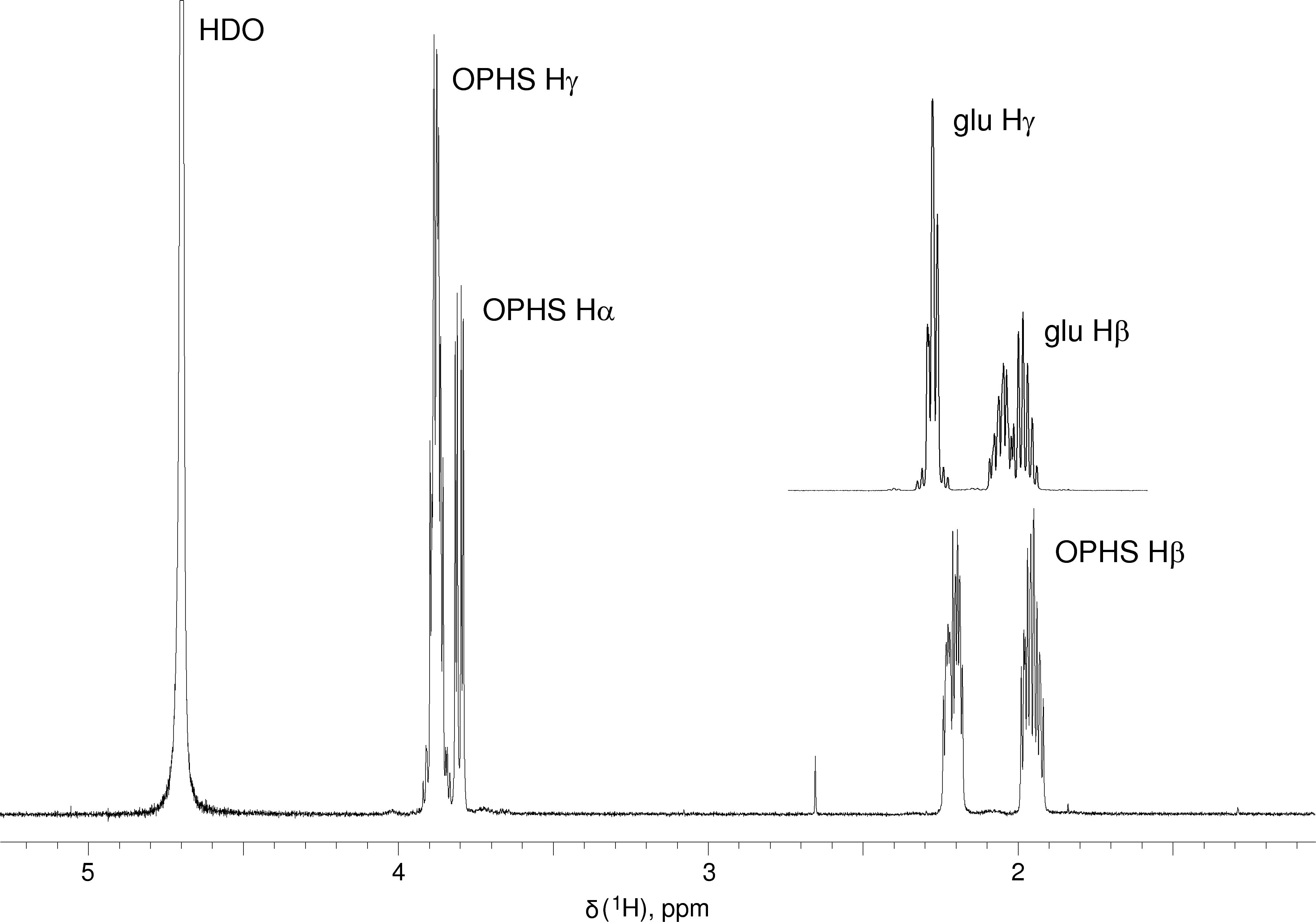


Figure S5. ^1^H NMR spectrum of OPHS. Reactions 1 and ‘2-3’ were performed in H_2_O with natural abundance reagents and the resulting OPHS was purified on a Q-sepharose FF column. Note that the transamination of 2 to 3 (part of reaction ‘2-3’) requires Glu that is subsequently converted to 2-ketoglutarate (Figure 2) and it is desirable to remove these compounds to ensure that extra protons do not get added during growth of highly deuterated proteins. The 1D spectrum shows that after purification of the ‘2-3’ reaction mixture by chromatography Glu and 2-ketoglutarate are removed. This can be seen by comparing the top trace showing the Hβ/Hγ region of Glu with the full spectrum (of OPHS) and by noting the absence of peaks at 2.8-2.9 ppm corresponding to the position of Hβ of 2-ketoglutarate (see Figure S3).


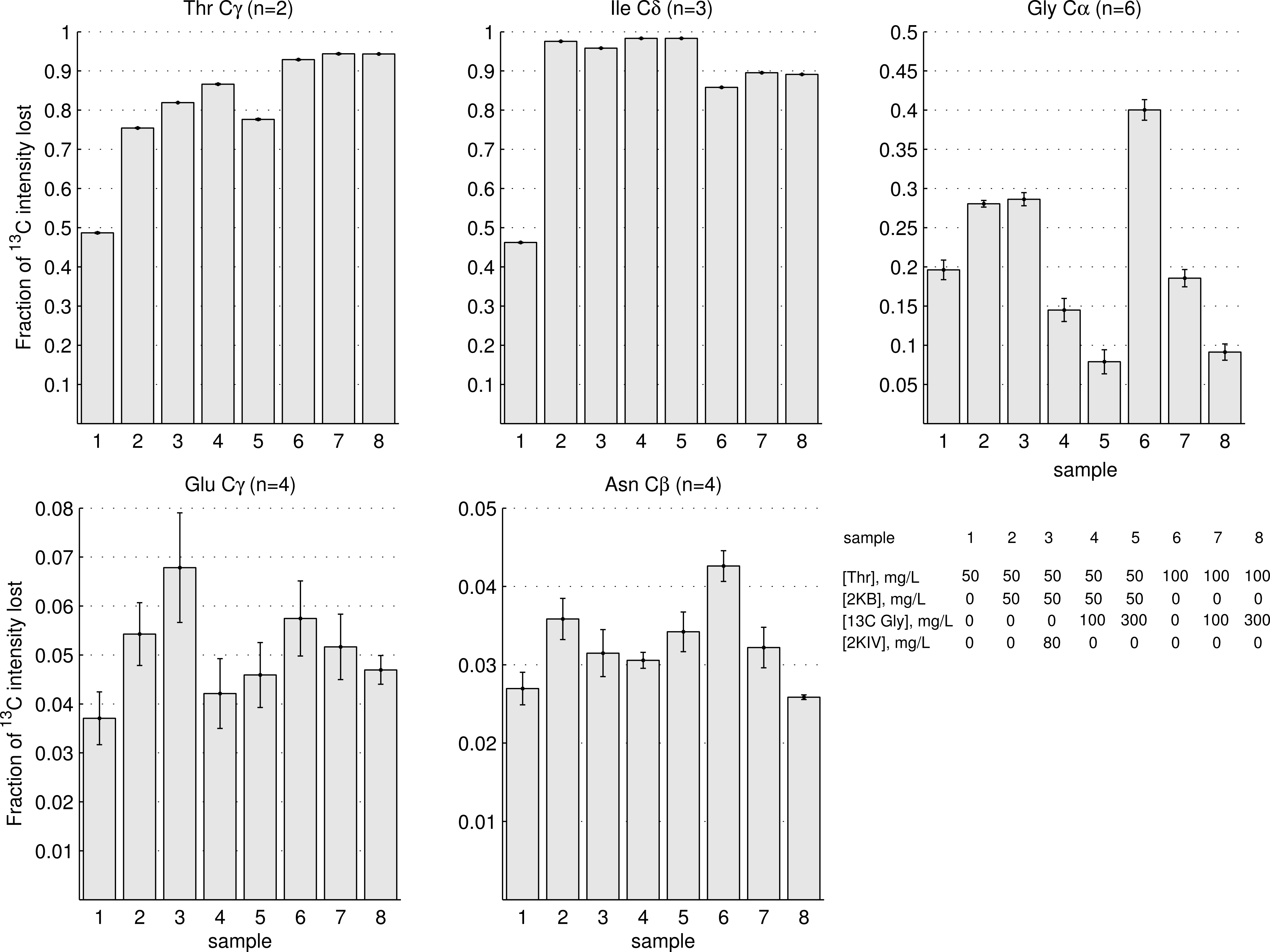


Figure S6. Fraction of ^13^C intensity lost at Thr Cγ, Ile Cδ1, Glu Cγ, Gly Cα, Asn Cβ positions of a U-[^13^C,^1^H] labeled Abp1p SH3 domain sample where ^12^C labeled precursors such as Thr, 2-ketobutyrate or 2-ketoisovalerate (2KIV) are added prior to induction of protein expression in the amounts indicated at the bottom of the figure. The value of ‘n’ corresponds to the number of residues of a particular type that are averaged over in each measurement, with the error bars denoting the range of measurement values. Histograms for Thr, Ile and Glu are as in Figure 4 of the main text and are included for ease of comparison. Results for Asn serve to monitor the diversion of added label into the TCA cycle.


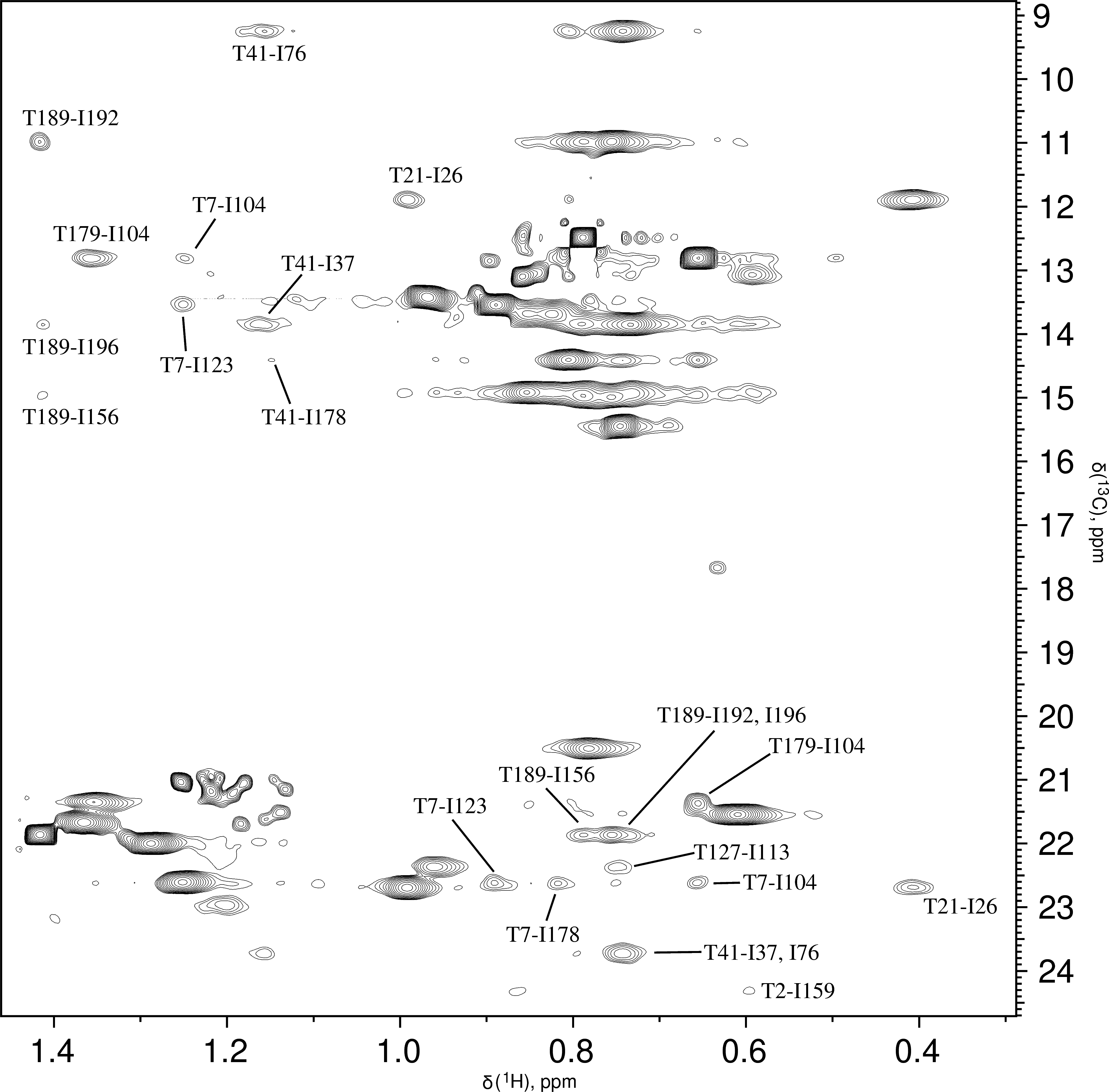


Figure S7. Annotated 2D, ^13^C,^1^H NOESY spectrum (250 ms mixing time) recorded on a 0.5 mM sample of U-[^2^H], Ile-δ1[^13^CH_3_], Thr-γ2[^13^CH_3_] α_7_β_7_β_7_α_7_ with labeling confined to the β rings (see text).

**References:**

1. Chen GJ, Qiu N, Karrer C, Caspers P, Page MG (2000) Restriction site-free insertion of PCR products directionally into vectors. Biotechniques 28: 498-505.

2. van den Ent F, Löwe J (2006) RF cloning: a restriction-free method for inserting target genes into plasmids. J Biochem Biophys Methods 67: 67-74.

3. Blackwell JR, Horgan R (1991) A novel strategy for production of a highly expressed recombinant protein in an active form. FEBS Lett 295: 10-12.

4. Murakawa T, Machida Y, Hayashi H (2011) Product-assisted catalysis as the basis of the reaction specificity of threonine synthase. J Biol Chem 286: 2774-2784.

5. Stollar EJ, Garcia B, Chong PA, Rath A, Lin H, Forman-Kay JD, Davidson AR (2009) Structural, functional, and bioinformatic studies demonstrate the crucial role of an extended peptide binding site for the SH3 domain of yeast Abp1p. J Biol Chem 284: 26918-26927.
